# Development of a new barcode-based, multiplex-PCR, next-generation-sequencing assay and data processing and analytical pipeline for multiplicity of infection detection of *Plasmodium falciparum*

**DOI:** 10.1186/s12936-021-03624-2

**Published:** 2021-02-16

**Authors:** Rebecca M. Mitchell, Zhiyong Zhou, Mili Sheth, Sheila Sergent, Michael Frace, Vishal Nayak, Bin Hu, John Gimnig, Feiko ter Kuile, Kim Lindblade, Laurence Slutsker, Mary J. Hamel, Meghna Desai, Kephas Otieno, Simon Kariuki, Ymir Vigfusson, Ya Ping Shi

**Affiliations:** 1 Division of Parasitic Diseases, Center for Global Health, Centers for Disease Control and Prevention (CDC), Atlanta, USA; 2 Department of Computer Science, Emory University, Atlanta, USA; 3 School of Nursing, Emory University, Atlanta, USA; 4 Biotechnology Core Facility Branch, Division of Scientific Resources, CDC, Atlanta, USA; 5 Office of Infectious Diseases, National Center for Emerging and Zoonotic Infectious Diseases, CDC, Atlanta, USA; 6 Liverpool School of Tropical Medicine, Liverpool, UK; 7 Kenya Medical Research Institute, Centre for Global Health Research, Kisumu, Kenya

**Keywords:** *Plasmodium falciparum*, Transmission, Multiplicity of infection, Haplotype and strain, StrainRecon, MOI estimation, STIM

## Abstract

**Background:**

Simultaneous infection with multiple malaria parasite strains is common in high transmission areas. Quantifying the number of strains per host, or the multiplicity of infection (MOI), provides additional parasite indices for assessing transmission levels but it is challenging to measure accurately with current tools. This paper presents new laboratory and analytical methods for estimating the MOI of *Plasmodium falciparum*.

**Methods:**

Based on 24 single nucleotide polymorphisms (SNPs) previously identified as stable, unlinked targets across 12 of the 14 chromosomes within *P. falciparum* genome, three multiplex PCRs of short target regions and subsequent next generation sequencing (NGS) of the amplicons were developed. A bioinformatics pipeline including B4Screening pathway removed spurious amplicons to ensure consistent frequency calls at each SNP location, compiled amplicons by SNP site diversity, and performed algorithmic haplotype and strain reconstruction. The pipeline was validated by 108 samples generated from cultured-laboratory strain mixtures in different proportions and concentrations, with and without pre-amplification, and using whole blood and dried blood spots (DBS). The pipeline was applied to 273 smear-positive samples from surveys conducted in western Kenya, then providing results into StrainRecon Thresholding for Infection Multiplicity (STIM), a novel MOI estimator.

**Results:**

The 24 barcode SNPs were successfully identified uniformly across the 12 chromosomes of *P. falciparum* in a sample using the pipeline. Pre-amplification and parasite concentration, while non-linearly associated with SNP read depth, did not influence the SNP frequency calls. Based on consistent SNP frequency calls at targeted locations, the algorithmic strain reconstruction for each laboratory-mixed sample had 98.5% accuracy in dominant strains. STIM detected up to 5 strains in field samples from western Kenya and showed declining MOI over time (*q* < 0.02), from 4.32 strains per infected person in 1996 to 4.01, 3.56 and 3.35 in 2001, 2007 and 2012, and a reduction in the proportion of samples with 5 strains from 57% in 1996 to 18% in 2012.

**Conclusion:**

The combined approach of new multiplex PCRs and NGS, the unique bioinformatics pipeline and STIM could identify 24 barcode SNPs *of P. falciparum* correctly and consistently. The methodology could be applied to field samples to reliably measure temporal changes in MOI.

## Background

Malaria infection remains a major public health problem in sub-tropical and tropical areas. *Plasmodium falciparum* is responsible for most malaria-attributed morbidity and mortality [1]. Accurately and timely measuring of the change in *P. falciparum* transmission levels is not only important in interpretation of data from epidemiological investigations and transmission-reducing intervention studies, but also is essential in the impact evaluation of programmatic activities on malaria transmission. Traditionally, the entomological inoculation rate (EIR) has been used for measuring malaria transmission level mostly for epidemiological studies [2]. However, EIR is not suitable for obtaining estimates of transmission level rapidly and its accuracy has been questioned in some studies [3–5]. Epidemiological approaches, such as cohort infection incidence studies, parasite prevalence surveys and passive case incidence data, are frequently used to measure transmission levels, but cohort incidence studies with relatively high precision are expensive and time consuming, and other malaria metrics may be subject to a number of biases [3]. Moreover, several malaria metrics could exhibit non-linear scaling relationships [3]. In pioneering work, measuring the multiplicity of infection (MOI, defined as the number of concurrent parasite strains per parasite-positive host) using molecular genotyping tools showed that MOI positively correlates with endemicity [6–8] and has been considered and proposed as an adjunct metric for characterizing malaria transmission [3, 9]. MOI can be the result of multiple mosquito bites (superinfection), a single mosquito bite (co-transmission) [10, 11], or both. The MOI metric, assuming adequate precision, can uncover parasite strain populations that may enhance understanding of transmission dynamics [3].

Previously established molecular assays, including size-based polymorphic antigenic genes or neutral microsatellites, have limited power to measure MOI accurately because they cannot determine the true parasite haplotypes within a host [12–14]. Although antigen-targeted or non-antigen-targeted gene (amplicon) deep sequencing could produce the information on gene-specific MOI and improve the sensitivity of minor variant detection [14–16], a single-target, deep sequencing strategy could not reflect the genomic signatures of parasite strains due to only a small genomic region sequenced. In addition, the gene-specific MOIs generated from multiple-target gene deep sequencing could vary even within a study [14, 16], depending on the different multiple targets chosen, the level of host immune pressure on specific antigen, and/or the different extent of diversity in the multiple targets either antigenic or non-antigenic genes.

Whole genome deep sequencing has improved MOI estimation at the population level by detecting genomic signatures and minority strains of parasites through newly developed analytical tools, StrainRecon [17] and DEploid [18]. The main difference between StrainRecon and DEploid is that DEploid requires a reference panel of strains to be provided as a prior for potential haplotypes present in the sample, whereas StrainRecon, discussed below, requires no templates or priors. Yet whole genome sequencing imposes practical challenges, particularly with respect to the large volume of parasite-infected red blood cell sample that is needed, along with time and cost, which together diminish the feasibility for rapid MOI estimation.

Separately, a molecular barcoding tool for identification and tracking of *P. falciparum* using 24 single nucleotide polymorphism (SNP) markers was developed by Daniels et al. [19] as stable, unlinked targets across 12 of the 14 chromosomes within the *P. falciparum* genome. Whereas this 24 SNP individual TaqMan real-time PCR barcode tool is used successfully in pre-elimination or low-endemic areas for detection of a unique fingerprint or signature for a parasite genome [20], it fails in environments where individuals have infections with multiple strains since the previously established laboratory and analytical tools cannot classify haplotypes and provide quantitative information on the number of strains within a host [19, 21]. The main objective of this study is therefore to overcome the obstacles in using the identified 24 SNP barcodes for MOI analysis in medium/high transmission areas. Here, the laboratory and algorithmic challenges of performing 24 SNP barcode-based MOI estimation suitable for medium/high transmission areas are addressed above. To this end, a complete pipeline for estimating MOI from blood was built, including molecular tools and numerical algorithms to determine likely barcodes, haplotype and strain number within an individual sample, resulting in a tool that can be useful across different transmission levels.

Described below is the development and validation of an advanced laboratory assay and unique data processing pipeline with B4Screening pathway for strain disambiguation using three multiplex PCRs followed by MiSeq deep sequencing based on the published panel of 24 SNPs of *P. falciparum* [19]. Further validation of MOI estimation was conducted on field samples collected over time from Kenya using the recently published algorithm StrainRecon [17] and a novel threshold-calibrated MOI estimation method, StrainRecon Thresholding for Infection Multiplicity (STIM) is presented below. The methods developed in this study offer malaria researchers the ability to target multiple genetic loci in sufficient depth to link across sites based on frequency of SNP reads at each site using the non-template approach. The consistency of these SNP frequencies determines the ability of the algorithm to successfully assign frequencies to haplotypes and disentangle samples that comprise of multiple strains [17].

## Methods

Since many different analytical methods are involved in the scope of this study, relevant details around each analysis method are provided in the corresponding section where appropriate. In addition, stepwise workflow is described in each technical section.

### Ethical considerations

The de-identified testing for Illumina deep sequencing at the CDC Malaria laboratory was determined as non-human subjects research by US CDC. The study protocols, from which field samples were obtained, were reviewed and approved by the Ethics Review Committee of the Kenya Medical Research Institute including blood sample collection and use of the samples for parasite genotyping.

### Development of multiplexing PCRs with next generation sequencing assay

#### Parasite strains and quantification

Six laboratory-cultured *P. falciparum* strains (D6, D10, 7G8, RO33, V1/S, W2) representing 24 known barcode SNPs were used for assay development. Extraction of DNA from cultured parasites was performed with QIAamp DNA Mini Kit (QIAGEN, Germantown, MD, USA). DNA was quantified using a real-time PCR protocol described by Daniels et al*.* [19] with a series of diluted standard curve (10^2^–10^5^) of the plasmid targeting a single copy of *Pf07_0076* gene.

#### Preparation of mixed parasites strains

Unlike the detection limit in traditional PCR diagnosis, the lowest limit for detecting a minor strain in a mixed strain infection is compounded by both lowest proportion of the strain and lowest parasite concentration (density). Citrated O^+^ whole blood was used for dilution of above quantified samples. Blood was spiked with 10^5^ parasites/µl with 3 different strain combinations (A: D10/D6/V1-S, B: D6/RO33/W2, C: 7G8/V1-S/R033). Each combination was prepared to target in 3 different proportions (1: 97.5–2–0.5%, 2: 95–4–1%, 3: 88–10–2%, respectively). These 9 combination-proportion preparations were then diluted in tenfold dilution series to 10^2^ parasites/µl. Based on the designed lowest proportion (0.5%) and lowest parasite concentration (10^2^ parasites/µl) described above, a target lowest limit for minor strain detection was expected at 0.5 parasite/µl. Each dilution stage was preserved as dried blood spots (50 µl per spot) on Whatman 903 filter paper (DBS) (GE Healthcare, Westborough, MA, USA) and 50-µl liquid aliquots in 1.5 ml sample tubes. The liquid aliquots were immediately frozen at − 80 °C. The filter paper spots were dried overnight in a biosafety hood and were then stored at − 80 °C in a Ziploc bag with 4 desiccant packs (IMPAKCorp., Los Angeles, CA, USA) and a moisture indicator. A single DBS (50 µl) and equally a 50 µl of liquid aliquot were used for each DNA isolation using QIAamp DNA Mini Kits (QIAGEN).

#### Multiplex PCR development

The primers for 24 SNPs by individual real-time PCRs described by Daniels et al*.* [19] were used for 3 multiplex PCR reactions with the addition of standard 16S sequencing overhang adaptor sequences [Forward overhang: 5′ TCGTCGGCAGCGTCAGATGTGTATAAGAGACAG‐(locus-specific primer) and Reverse overhang: 5′ GTCTCGTGGGCTCGGAGATGTGTATAAGAGACAG‐(locus-specific primer)] [22] for use in MiSeq library preparation. Multiplex combinations were designed via PrimerSelect (DNAStar Lasergene, Madison, WI, USA) to minimize primer-dimers (artifacts) in each multiplex reaction. Multiplex combinations were 7-way (SNPs: 2, 3, 6, 8, 10, 15, 23); 8-way (SNPs: 4, 5, 7, 11, 12, 14, 16, 21) and 9-way (SNPs: 1, 9, 13, 17, 18, 19, 20, 22, 24). For each multiplex reaction, a stock primer solution was made of all forward and reverse primers with a concentration of 100 µM each.

Multiplex PCR reaction contained 12.5 µl 2× master mix Platinum Multiplex PCR supermix (ThermoFisher Scientific, Waltham, MA, USA), 100 nM each primer, 1 µl DNA template and PCR water in a 25-µl total reaction volume. Samples were run with 2 min of initial denaturation at 95 °C, followed by 35 cycles of 30 s denaturation at 95 °C, 1 min and 30 s annealing at 60 °C and 30 s extension at 72 °C, with a final extension at 72 °C for 10 min and final resting temperature of 4 °C.

Following visualization via DNA electrophoresis on a 2% agarose gel with 3 µl of 50 bp Track-it ladder (ThermoFisher Scientific) to ensure amplification, the short PCR products of 3 multiplex PCRs were pooled together for a sample and purified collectively on QIAquick PCR purification columns (QIAGEN). The purified DNA was quantified using a Nano Drop 2000 instrument.

#### Testing with pre-amplification (two-step PCRs)

To evaluate the ability to amplify low frequency SNPs, a comparison test between one-step multiplex PCR described above and a pre-amplification PCR step before multiplex PCR with PreAmp PCR kits (ThermoFisher Scientific) was conducted. The pre-amplification step used the same primers as each individual multiplex at 100 nM each, with 10 µl of TaqMan Preamp Master Mix solution, 1 µl DNA template and PCR water up to 20 µl total reaction volume. Following pre-amplification, products were diluted 1:20 and 1 µl diluents were carried over into the multiplex PCRs described above.

#### Testing with different concentration in laboratory-cultured strains

To evaluate the minimum detection threshold of multiplex PCRs and the NGS (see below for detailed NGS), serial dilutions of *P. falciparum* at 10^2^ to 10^5^/µl from the different combinations and proportions of parasite strains were tested in triplicate. All samples with parasite concentration < 10^5^/µl were run both with and without a pre-amplification.

#### Testing with different type of samples using laboratory-cultured strains

Both frozen liquid blood samples (50 µl per sample) and DBS samples (50 µl per spot) were tested with and without pre-amplification. In total, final 108 samples generated from laboratory strains (with different combinations, proportions, concentrations, and sample types) were tested.

#### Testing with field samples

Two-hundred and seventy-three smear-positive samples from 4 cross-sectional surveys in Asembo, western Kenya were used for this study [23–25]. Among these, 65 samples from 1996 and 72 samples from 2001 were randomly selected in children from 6 months to 5 years, and 53 samples from 2007 and 83 samples from 2012 were used based on the availability of samples in individuals from 6 months to 20 years. Since age is known not to affect parasite diversity or MOI measurements when neutral markers, such as microsatellites and 24 SNP barcode, are used [20, 23, 26], it was expected that the 24 SNP barcode-based MOI measured in this study is also unlikely to be influenced by age [20, 26]. The field samples were tested in the same multiplex PCR and NGS conditions as the laboratory-cultured mixed parasite strains with or without the pre-amplifications. Laboratory-cultured strains as positive controls and normal blood as negative control were used for each experimental run for the field samples.

#### MiSeq library preparation and run

MiSeq Libraries were prepared using a standard 16S Metagenomics sequencing protocol (Illumina) [22]. Briefly, the PCRs for attaching sequencing indexes and adapters were performed with the Nextera XT Index Primers with dual index barcodes followed by a cleaning process. The final libraries were analysed on Fragment Analyzer for size and concentration (ng/µl). The libraries for all the samples were normalized to 10 nM by diluting in elution buffer and pooled at equal volume. The final 10 nM pool was diluted to 2 nM and denatured and diluted for loading on the Miseq flowcell. Up to 54 barcoded samples were pooled in one library (per plate) using Illumina 2 × 250 bp run. On completion, sequence reads were filtered for read quality, base called and demultiplexed using bcl2fastq (v2.19). The sequencing results were saved for bioinformatics analysis.

### Development of bioinformatics analysis pipeline for processing NGS data

The bioinformatics analysis pipeline developed in this study was to clean NGS data for consistent frequency calls at each SNP site across 12 chromosomes. Because 24 SNP barcodes differ from 16S metagenomics data in depth of coverage and amplicon diversity, an existing 16S bioinformatics pipeline was modified, and several additional data cleaning components were integrated as described below. This novel pathway, called B4Screening, involves four steps: initial adaptor trimming and cleaning, Bioconductor dada2 pathway, targeted removal of mismatched primers or probes, and a random forest classification of remaining reads. All steps are detailed below.

#### Data processing

Sequences were first trimmed to eliminate Illumina adaptor primers by CutAdapt 1.8 and cleaned by Prinseq 0.20.3. Quality was examined via FaQCs 1.34. Following CutAdapt and Prinseq, sequences were loaded into R to use a modified Bioconductor 16S pipeline. Dada2 (v1.6.0) was used for secondary filtering and trimming, error rate detection, pairing, and chimera removal. Following processing in the dada2 pipeline to remove bimeras, a second chimera removal step was performed which required that forward and reverse primers matched the same target location. The first and last 15 base pairs of each read were compared to the lookup table of potential primers, and the best matching primer (maximum of 2 mismatches) was identified as the input primer. Reads were reverse complimented and those reads without a matching forward and reverse primer pair were removed. Matching to initial probe location from the publication [19] was used to evaluate whether reads matched the reference or alternative base at the SNP site. If neither reference nor alternative SNP (as designated binary) matched, the reads were removed. For each amplicon, reference and alternative probe sequences were trimmed to the identical length, and number of mismatches was calculated for each probe. Amplicons that had more than two mismatches to both probes were discarded as a spurious amplicon. Because the database included SNPs only, amplicon length variations greater than one base pair *versus* expected reference length were removed. The low frequency variants (< 0.1% of the total sequences) were also removed.

#### Matching to reference sequences

The Bioconductor pipeline was designed for 16S data, therefore, the assignment of genus and species was used as proxy for amplicons matching the correct targets in the genome. Each region was entered as part of a reference library. Genus level assignment was provided via assignTaxonomy with a minimum bootstrap value of 80%. Reference amplicon sequences from the *P. falciparum* 3D7 were identified as exact matches for “species” assignment in dada2 assignSpecies. A phyloseq object was produced (Phyloseq version 1.22.3) incorporating SNP site and other relevant sample information and unique amplicons for all possible variants in the dataset prior to removal of low frequency amplicons.

#### Designing a random forest (RF) machine learning classifier

To address the amplicons generated by multiplex processing in data in which sample composition is not known, a classifier was trained on 3/4 portion of the data and tested on the remaining quarter. This classifier was generated in caret (v 6.0.84 in R) using a random forest (RF) design. The classifier was provided with 4 elements: (1) proportion of all unique amplicons by sample; (2) proportion of all unique amplicons by plate; (3) reads with SNP by plate; and, (4) sample reads per plate, all of which should be reflective of unique amplicon distribution in field data. Repeated tenfold cross validation (10, repeat 3) and features were evaluated in both the laboratory strain dataset and datasets in which sample characteristics are unknown. As a test, the classifier was also trained on two combinations and evaluated on a third for comparison of unrelated data.

Initial amplicon training labels were generated based on mixes of laboratory strains with known SNP locations. Training labels of “positive” and “negative” were based on three criteria. For a positive label: (1) strains had to be present in more than 12 samples of the 36 samples representing each combination; (2) strains had to be more than 0.1% of the data at that SNP location; and, (3) strains had to change frequency with changing proportions of strains in models accounting for expected frequency ratio with MiSeq run as a random effect (using lmer in R). Failing to meet any of the above criteria resulted in a negative label.

#### Read depth and frequency determination

Following removal of all spurious amplicons, only samples with at least 16 SNP sites represented with a read depth of at least 500 for each SNP were retained for frequency estimation. Read frequency for reference (REF) versus alternative (ALT) alleles was calculated by aggregating all confirmed amplicon sequence variants within a genus designation by target SNP. To determine the influence of preprocessing and parasite density on reads per locus, log_10_ reads per locus were modelled using random effects mixed models in R (lmer). Formula: ReadsPerLocus = ParasiteConcentration + PreAmplification + Proportions + Mixture + Plate, with a random intercept: Sample.

#### Consistency of read frequencies

To get initial point estimates for SNP read frequencies in each experimental combination-mixture pairing, restricted regression (R package restriktor) was performed per unique experimental combination. Frequency calls within each sample were analysed for impact of pre-amplification or parasite concentration while controlling for unique biologic mixture (combination, proportion, sample type) using a linear model.

### Strain reconstruction and haplotype analysis

A previously developed mathematical algorithm, StrainRecon [17], was used for identifying haplotypes and enumerating strains based on frequency results of individual 24-SNP barcodes generated from NGS. Briefly, the basic StrainRecon algorithm accepts a vector ${\varvec{s}}$ of SNP frequencies as input and a parameter ***n*** denoting the anticipated number of strains. The output is twofold: a binary matrix ***M*** containing ***n*** reconstructed strain barcodes, and a vector ***v*** of strain mixtures. The strains found by the algorithm represent the maximum likelihood estimate of the original barcodes under three assumptions. First, the overall error from all steps of the pipeline can be modelled as normally distributed (Gaussian) noise. Second, noise values are independent between SNP sites. Third, the metric minimized to optimize the solution quality is $Mv - s_{2}$, called the *misfit*. (Here, $\left\| \cdot \right\|_{2}$ denotes the standard Euclidean L2-norm.) Both ***M*** and ***v*** are assumed to be completely unknown, making this a mathematical inverse problem. StrainRecon models the problem as a Bayesian maximum-a-posteriori (MAP) estimation problem, using block coordinate descent to quickly converge at a solution when the number of strains *n* is small. The algorithm further provides an average (and standard deviation) over the entire posterior distribution of candidate solutions, which can be used to illustrate confidence or ambiguity of each call the algorithm makes within a barcode or strain mixture fraction. In this paper, the StrainPycon 1.0 [27] implementation of the StrainRecon algorithm for Python 3.4.3 was used for analysis. Each sample was evaluated for barcode sequences given the assumption that field samples can include up to and including 6 strains.

### StrainRecon thresholding for infection multiplicity (STIM) algorithm

StrainRecon allows discrimination of the pattern of haplotypes that are sufficiently prevalent in a sample relative to the experimental noise, as measured by misfit. The goal of this study was to create a tool that can further estimate the true number of parasite strain infections in a sample without the need for templates while being aware that extremely low-proportion strains in the sample are indistinguishable from noise. The method, therefore, takes each sample and runs the StrainRecon algorithm in a loop over number of strains *n* to determine the misfit of the MAP solution for each *n*. As the number of free parameters increases with *n*, the largest misfit will be found with *n* = 1, and the misfit decreases with larger *n*. In lay terms, it will always be possible to better fit the strains of a sample by imagining that it contained more haplotypes, but some of these haplotypes may be an artifact of introduced variation inherent in NGS sequencing. The approach used in this study for determining MOI was to set a threshold value, called *T*, on the misfit value and to return the lowest *n* whose MAP estimate had misfit below *T*. Together, the complete procedure is referred to as the STIM algorithm.

The upper bound of *n* is limited by noise levels in the analytical pipeline. In practice, when error rates are in the range of 1–3%, the upper bound of *n* that can be meaningfully processed is about 5–6 strains. Specifically, even under optimistic assumptions about mixture proportions for strain disambiguation (which is when mixture proportions are proportional to the first powers of 2), an inverse problem barring an informative Bayesian prior or a tailored noise model will be unable to differentiate more than $n = - \log_{2} \left( \varepsilon \right)$ strains from noise level of $\varepsilon > 0$. For example, at 1% error, this bound is $n \le - \log_{2} \left( {0.01} \right) \approx 6.6$. Within these constraints, the STIM method has the crucial advantage of estimating MOI while not depending on any template information, unlike DEploid [18], for example.

## Results

### Sequencing coverage in laboratory strain samples

Of the 23.48 million Prinseq-cleaned, paired reads in the 108 laboratory-generated samples, 21.47 million reads remained prior to evaluating with the RF classifier (see next subsection for development of RF classifier). The number of unique amplicons assigned (across all 24 loci) in the laboratory strain dataset dropped from 9901 following the standard dada2 pipeline to 360 following additional filtering for primer mismatch and probe mismatch. Potential unique amplicon numbers per SNP target location in this screened set were varied, with a minimum of 2 (SNP14, SNP18) and a maximum of 70 (SNP11).

**Fig. 1:**
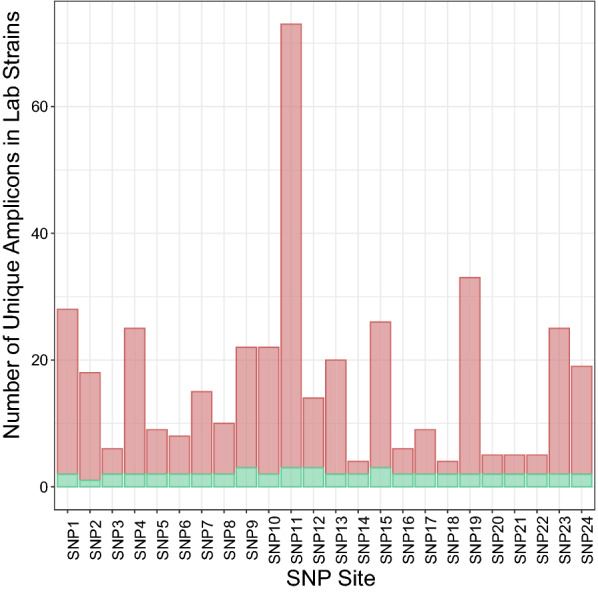
Distribution of unique amplicons in laboratory strains. Red bar represents number of unique amplicons following dada2 Bioconductor processing steps of B4Screening and additional matching-based steps, and green bar shows number of unique amplicons following RF classifier B4Screening steps, separated by SNP target location

Of these 360 unique amplicons, 51 were retained following RF classification, and represented the expected SNPs at the target locations (Fig. 1) and several additional SNPs at different locations in the amplicon. The final number of unique amplicons per SNP target location ranged from 1 to 3 in the laboratory strain data. Overall, 21.35 million (90.9%) reads remained following exclusion of spurious amplicons via the RF classifier (see Additional file 1: Fig. S1). For each location, unique amplicons retained after RF classification were summed by target SNPs to generate frequencies for StrainRecon.

### RF machine learning classifier development using laboratory strains

There were 5193 unique datapoints for classifier development in the laboratory strain dataset, accounting for 2576 of the 2592 potential SNP sites among all 108 samples (not all SNPs had > 500 reads in each sample). If the classifier was trained with 75% of the total data selected, 3881 data values were used for training and 1312 for testing.

In the 1312 test set, all 1161 positively coded unique amplicons were correctly identified and all 151 negative unique amplicons as initially coded were also correctly identified. Alternatively, if a single mixed combination C was withheld as a test set rather than a random selection of unique amplicons across all data, to protect against inadvertent data leakage, 3369 data-samples combinations were used for training and 1824 for testing. In this case 1587 out of 1624 positively coded unique amplicons were correctly classified (sensitivity: 97.7%) while 197 of 200 (specificity: 98.5%) negatively coded unique amplicons were correctly classified. Overall, the amplicons classified for removal were low copy number relative to the total representation of the target region (see Additional file 1: Fig. S1).

When the field samples from western Kenya were processed, the same RF classifier was used to screen viable amplicons.

### Read depth and coverage of target SNP sites in laboratory strains

The proportion of samples retaining sufficient read depth across all SNP locations following RF classification was evaluated. A cut-off of 500 reads was selected to ensure that precision would not be influenced by read depth. With a minimum coverage depth of 500 reads post-processing, SNP 16 was absent in 16 of laboratory samples (15%). No laboratory strain samples had fewer than 16 total SNPs represented, and while the fifth percentile number of SNPs present within a sample was 21, 78 of 108 samples had all 24 SNPs present. With a cut-off of 500 reads, individual SNP read depth was lowest for SNP18 (2090 median read depth), and highest for SNP10 (17,630 median read depth) (see Additional file 2: Fig. S2).

### Relationship between read depth and sample characteristics

**Fig. 2:**
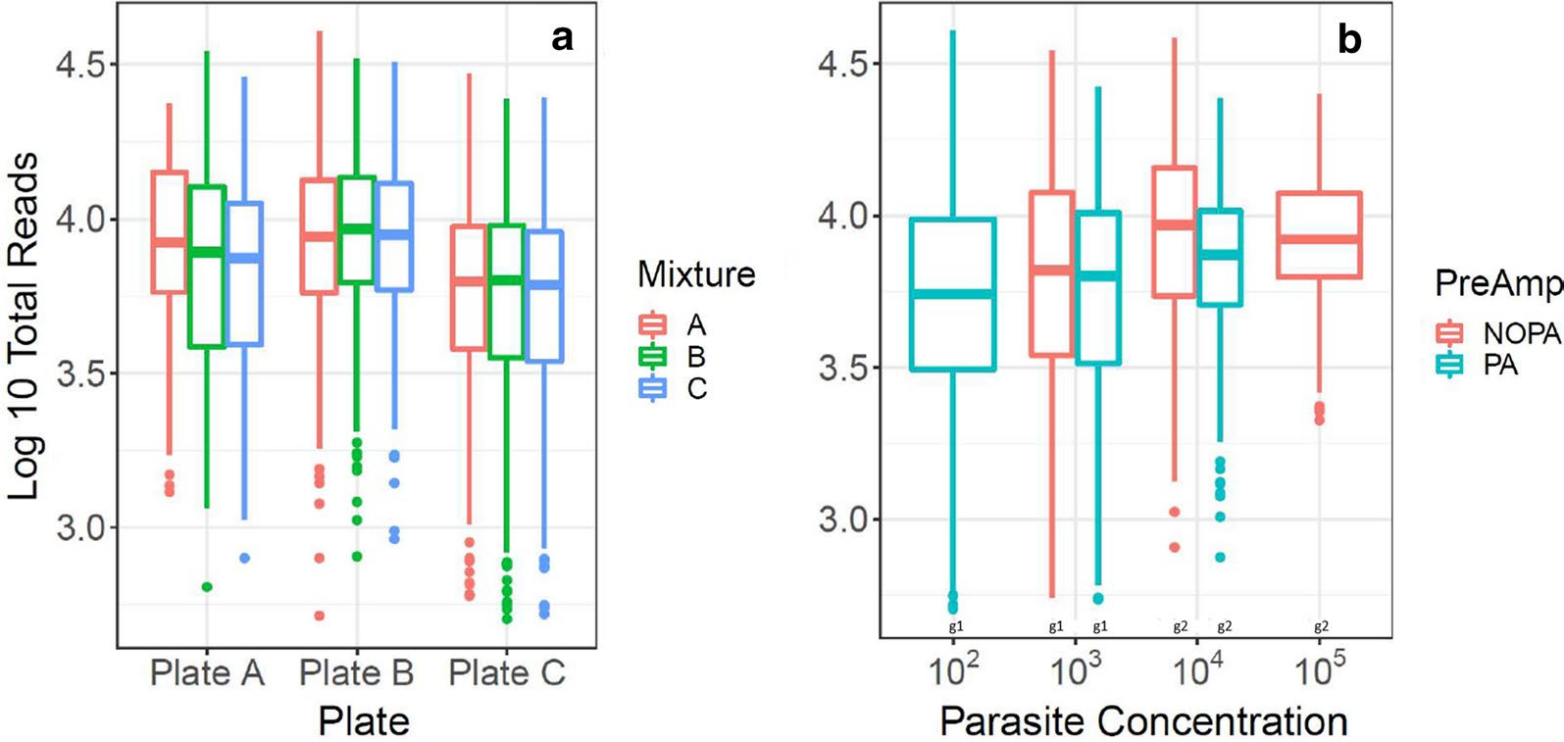
Relationship between read depth and sample characteristics in laboratory strain mixes. **a** Reflects log_10_ total reads by plates that contain 3 different strain mixtures A: D10/D6/V1-S, B: D6/RO33/W2, C: 7G8/V1-S/RO33 respectively, as cited in Methods. **b** Reflects impact of pre-amplification (PA) and non-pre-amplification (NOPA), separated by parasite concentration as not all parasite concentration/pre-amplification combinations exist in the data. The groups that are significantly different in read depth by original parasite concentration are indicated by “g1”and “g2” symbol, respectively

Multivariable models assessed the relationship between sample characteristics and read depth. Neither proportion of strains in a mixture (p = 0.43), nor specific strain mixture (p = 0.67) was associated with read depth (Fig. 2a). Figure 2a also showed a low variability among three different processing pipelines. Parasite concentration was non-linearly associated with average read depth [(10^2^ = 5500, 10^3^ = 6170, 10^4^ = 8310, 10^5^ = 8310 reads/site), p < 0.0001]. Samples with 10^2^ and 10^3^ parasite concentration did not differ from each other, nor did samples with 10^4^ versus 10^5^ parasite concentrations. However, comparisons among other pairs were significant (emmeans, Tukey correction, p < 0.01) (Fig. 2b). Read depth did not significantly differ between pre-amplified and non-pre-amplified samples [(6460 vs. 7410 average reads/site), p = 0.053] (Fig. 2b). Both DBS and frozen blood plates yielded sufficient reads in each SNP location.

### Consistency of read frequencies in laboratory strain samples

**Fig. 3:**
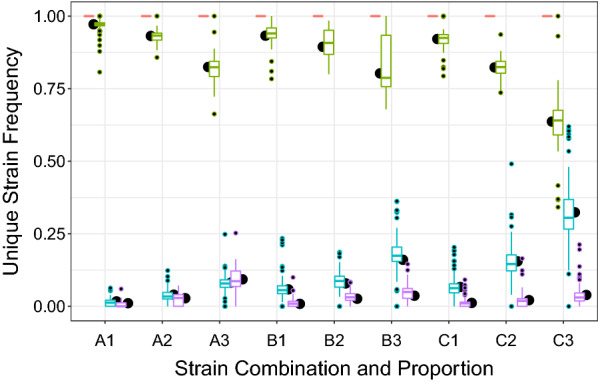
Frequencies of unique SNPs across all parasite concentrations, pre-amplification and sample types from laboratory strain mixes. Red is values with no unique strains at given SNP location, green is where the highest proportion strain is unique, teal where the intermediate proportion strain is unique and purple where the lowest proportion strain is unique. The estimated SNP frequency for each strain as generated in restriktor is represented in black dot. Combinations (A, B, C) were listed with proportions (1, 2, 3) as cited in Methods

SNP frequency calls for unique strains must be consistent within a single sample to ensure consistent barcode calls. Boxplots for frequencies across all parasite concentrations, pre-amplification and sample types (Fig. 3) show the full range and central tendency for unique-strain SNP frequencies across all conditions in laboratory strain data. A single site (SNP23) in Combination A mix was excluded from this analysis as the high proportion template appears to have both potential SNPs present in the original source material (see Additional file 3: Fig. S3).

Within a sample, a restricted regression linear model for proportion of reads matching the 3D7 reference sequence was fit with all three strains and categories (low, medium, high proportion), constrained to 1 (l m, and restriktor packages in R) to assess consistency across samples prior to running strain reconstruction. Unique strain frequencies were not influenced by pre-amplification (p = 1), or parasite concentration (p = 1), but differed by combination and strain proportion category (low/intermediate/high).

### Reconstruction of strain barcodes using laboratory strain mixes

Using SNP frequency calls, StrainRecon’s MAP estimates of 108 laboratory strain samples were calculated, varying the parameter *n* of the number of strains to be reconstructed (anticipated MOI) between 1 and 5. The reconstructed barcodes for each sample were compared to ground truth barcodes intended to be mixed, counting the number of reference/alternate matches at SNP sites with read depth of at least 500.

The 72 samples from Combinations B and C contain three strains. The dominant strain in the 36 samples from Combination A consistently generated two distinct reads at SNP23 (see Additional file 3: Fig. S3), indicating a likely presence of mutation. These two distinct reads at SNP23 were reconstructed independently, and Combination A samples were therefore characterized as containing four strains (33% of samples).

Table 1 shows that over 98.5% of the base pairs in the dominant “high” strains (target proportion range of 88–97.5%) were identified correctly, even when the algorithm is configured to identify more strains ($n \le 5$) than are truly mixed in the samples [17]. The “intermediate” strains (target proportion range of 2–10%) were reconstructed at 77.5–89.3% accuracy, and the “low” strains in the mixtures (target proportion range of 0.5–2%) were reconstructed at 47.1–64.6% accuracy. Among these, when using parameter n = 3 (true mixtures in samples), the accuracy for “high” strains, “intermediate” strains, and “low” strains were 98.7, 89.3 and 64.6%, respectively. Total DNA template material affected the ability to reconstruct barcodes, with percentage of correct SNP calls in the three true strains at 75.0% among all mixtures at 10^2^ parasites/µl, 80.6% at 10^3^ parasites/µl, 88.4% at 10^4^ parasites/µl, and 90.6% at 10^5^ parasites/µl.

**Table 1:** Barcode reconstruction quality with StrainRecon, varying the number of strains to be found

Proportion of SNP sites correctly reconstructed	Strain 1 (%)	Strain 2 (%)	Strain 3 (%)	Strain 4^a^ (%)
Anticipated MOI n = 1	98.97			
Anticipated MOI n = 2	98.97	84.79		
Anticipated MOI n = 3	98.70	89.25	64.64	
Anticipated MOI n = 4	98.78	82.69	48.80	19.79
Anticipated MOI n = 5	98.50	77.52	47.06	19.28

Columns are arranged by proportion of strain in the mixture, so the most dominant strain is called Strain 1. Non-barcode SNP sites are omitted

^a^Strain 4 exists only in those samples where SNP23 had a mutation

It is noted that larger *n* provides more free parameters, thus allowing solutions to have a better fit to the SNP frequency vector than smaller *n*, even when *n* exceeds the true number of strains in a sample. In this latter case, the algorithm “overfits” and attempt to explain experimental noise in the data with extra low-frequency strains to score a lower misfit value (see Additional file 4: Fig. S4 and Additional file 5: Fig. S5 with detailed information using individual sample examples). However, the third strain (and mutated fourth strain for Combination A samples) in all mixtures had very small proportion, even less than the estimated noise level of the experiment (≤ 5%), which deteriorates the barcode reconstruction.

### StrainRecon thresholding for infection multiplicity (STIM) algorithm

**Fig. 4:**
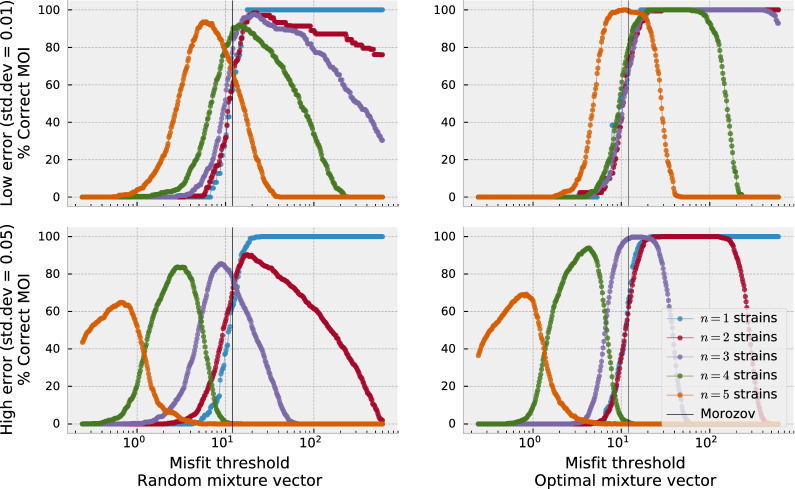
STIM estimation method on synthetic data. The data was generated by creating uniformly random barcodes and mixture vectors and adding Gaussian noise (top: std.dev = 0.05, bottom: std.dev = 0.01) to create input vectors for StrainRecon. The graph shows the percentage of synthetic samples for which the MOI was correctly identified by choosing the lowest number of strains that go below the misfit threshold on the horizontal axis. The plots on the left show regular performance, whereas on the right StrainRecon is provided with the correct value of the mixture vector. The black vertical lines show lowest misfit threshold permitted by Morozov’s discrepancy principle (here the expression simplifies to $\frac{SNPs}{2} = 12$), below which any signal is effectively better explained by noise [17]

Recall that the StrainRecon method takes the number of anticipated strains *n* as input and then determines what barcodes and mixture vectors of *n* strains produce the best fit (lowest misfit) by minimizing noise. A natural approach for estimating the true number of strains is then to place a threshold on the acceptable fitness level and determine how many strains *n* StrainRecon needs to produce barcodes and mixture vector with sufficiently low misfit. This algorithm is called STIM. The STIM estimation method was first evaluated on synthetic data produced by generating 1000 barcode matrices ***M*** and mixture vectors ***v*** uniformly at random for each specific number of strains *n* and calculating the dot-product ***Mv***. Following the StrainRecon framework [17], Gaussian noise with a known standard deviation ${\upgamma }$, corresponding to the amalgam of errors and reads in the laboratory pipeline, is added to the dot-product before the resulting vector is reconstructed. Figure 4 (left) varies the threshold value *T* on the horizontal axis, showing that lower noise levels give rise to misfit thresholds where MOI can be discerned for a wider range of strain counts. For comparison, Fig. 4 (right) shows how well STIM performs when, mathematically, the proportion of each strain in the mixture (vector ***v***) is ideally separated from others (by being proportional to powers of 2 as can be shown through mathematical analysis). The noise on the mixture values is normally distributed around a mean of 0 with standard deviation ${\upgamma }$ of either 0.05 (top row) or 0.01 (bottom row). Since normally distributed variables fall within two standard deviations from the mean 95% of the time, these ${\upgamma }$ values correspond to hypothetical pipelines whose accuracy for SNP reads are expected to lie with within 10 and 2%, respectively, of their true values at least 95% of the time. The black vertical bar shows the threshold value suggested by Morozov’s discrepancy principle, a point below which data are effectively best explained by noise. The results show that STIM is sensitive to noise levels: lower underlying error rate (standard deviation of 0.01) allows differentiation of strain count between 1 and 5 at some thresholds, whereas high underlying noise (standard deviation of 0.05) is more restrictive.

### Calibrating the STIM threshold

The assumption of Gaussian noise works well for in silico, but a rigorous model for experimental noise in practice is lacking. To adapt STIM for field data the first step was to calculate the MOI threshold *T* for laboratory strain mixture data (108 samples) while simultaneously calibrating MOI values that are analytically predetermined. To tolerate error in the pipeline and the noise-to-signal uncertainty in StrainRecon, the threshold *T* was varied while conservatively measuring whether samples from the laboratory strain experiment were called at $n = 3 \pm 1$ strains. The case for $n = 2$ accounts for mixture proportions that were so low (0.5–1%) relative to noise so that the corresponding samples effectively have two strains. Conversely, $n = 4$ accommodates the Combination A samples in mixtures that had a mutation in SNP23 in one of the laboratory strains, thus creating 4-strain combinations (Additional file 3: Fig. S3). In STIM, the calibrated threshold of $T = 1.8 \times 10^{ - 7}$ provided MOI within $n = 3 \pm 1$ strains for 101 out of 108 laboratory-mixed samples (93.6%). Overestimation (n = 5) was observed in 7 out of 108 laboratory samples (7.4%). The results are delineated in Table 2. To account for any potential inaccuracy in the threshold choice determination, the final step evaluated the field results across a range of threshold values (see Fig. 6).

**Table 2:** Distribution of MOI estimates by STIM on 108 laboratory-mixed samples using the threshold $T = 1.8 \times 10^{ - 7}$

True MOI	1	2	3	4	5
n = 4 (Comb. A)	0	3	13	*17*	3
n = 3 (Comb. B)	0	4	*21*	11	0
n = 3 (Comb. C)	0	3	*15*	14	4

Estimates for the true MOI value are italicized

### MOI in Kenyan field samples

The StrainRecon and STIM algorithms with a threshold of $T = 1.8 \times 10^{ - 7}$ were used to evaluate field samples from 4 cross-sectional surveys conducted in Kenya. A Kruskal–Wallis *H*-test (non-parametric one-way analysis of variance that extends the Mann–Whitney U test) on the MOI differing among smear-positive samples from 1996, 2001, 2007, and 2012 surveys was significant ($H = 29.7$, $p < 2 \times 10^{ - 6}$). The result suggests that the distribution of MOI in at least one of the years stochastically dominated another.

**Fig. 5:**
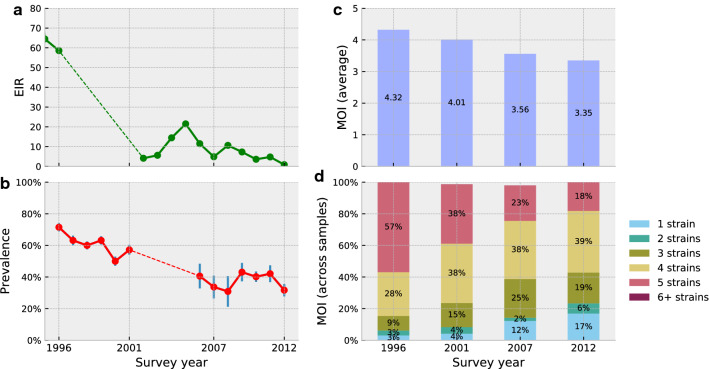
Metrics for assessing the change in transmission level in western Kenya. **a** EIR in Asembo, western Kenya from 1995 to 2012. **b** Malaria prevalence in age under 5 years old with a 95% confidence interval in same area from 1996 to 2012. **c** MOI as estimated by the STIM algorithm from 1996, 2001, 2007, and 2012 surveys in same area, presented as average MOI. **d** Percentage of samples with different number of strains in same area from 1996, 2001, 2007, and 2012 surveys. EIR data and malaria prevalence data, based on smear diagnosis, were extracted from published [38] and unpublished (KEMRI/CDC) data. The StrainRecon and STIM algorithms with a threshold of $T = 1.8 \times 10^{ - 7}$ were run for the SNP frequency data generated from smear-positive samples from 4 surveys in Kenya for MOI estimation

**Fig. 6:**
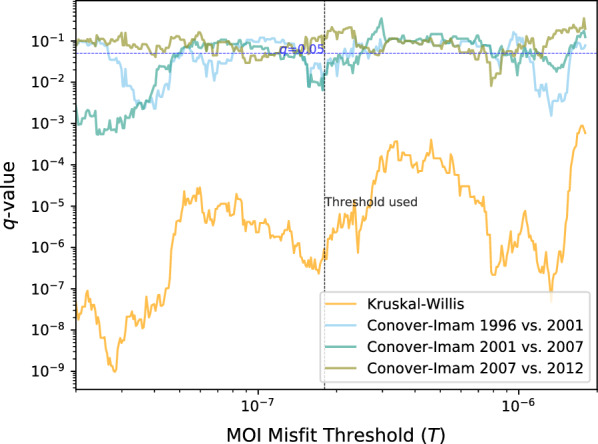
FDR-adjusted significance levels of the key statistical tests with different STIM thresholds *T*. Robustness across a neighborhood of values around the chosen *T*, shown on a log–log scale. The dotted black vertical line is the threshold derived from laboratory data and was used to generate Fig. 5c, d and Table 3; the dotted blue horizontal line is the significance level of $q = 0.05$. The strain count was not significantly different between 2007 and 2012 in the vicinity of the MOI misfit threshold used

To determine the differences in MOI between years, the Conover-Imam test was carried out to compare pairwise stochastic dominance for all pairs. False discovery rate (FDR) was controlled for using the two-stage step-up method of Benjamini, Krieger, and Yekutieli (BKR), which improves the power of the well-known Benjamini and Hochberg (BH) FDR mitigation method without making additional assumption [28]. The results with the FDR-corrected *p* values (*q* values) are shown in Table 3. The comparisons showed significant decrease in MOI ($q < 0.02$) between every pair of survey years between 1996 and 2012 with the exception of 2007 and 2012 ($q = 0.15$). In best fitting results from STIM, all samples contained either 5 or fewer strains and no sample contained 6 (or more) strains above the 5% noise level resolution of STIM. Notably, strains of low proportions (≤ 5%) in samples are indistinguishable from pipeline noise in the MOI estimates. The STIM results thus suggest a decline from an average of 4.32 strains per infected person in 1996 to 4.01, 3.56, and 3.35 in the years 2001, 2007, and 2012, respectively (Fig. 5c). Figure 5d also shows that the fraction of samples with one strain increased from 3% in 1996 to 17% in 2012 while the fraction of samples with 5 strains reduced from 57% in 1996 to 18% in 2012. The FDR-adjusted statistical test results for STIM are robust to changes in the misfit threshold parameter *T* (Fig. 6) except between 2007 and 2012 which straddles the $q = 0.05$ boundary.

**Table 3:** Difference in MOI across years in the Kenyan field samples

	1996	2001	2007	2012
1996		$1.7 \times 10^{ - 2}$*	$9.8 \times 10^{ - 5}$*	$4.4 \times 10^{ - 7}$*
2001	$1.7 \times 10^{ - 2}$*		$1.6 \times 10^{ - 2}$*	$7.0 \times 10^{ - 4}$*
2007	$9.8 \times 10^{ - 5}$*	$1.6 \times 10^{ - 2}$*		$1.5 \times 10^{ - 1}$*
2012	$4.4 \times 10^{ - 7 }$*	$7.0 \times 10^{ - 4}$*	$1.5 \times 10^{ - 1}$	

FDR-corrected *p*-values (*q*-values) of a Conover–Imam significance tests for difference in MOIs across all pairs of years in the Kenyan field samples. Significance at $q = 0.05$ level is denoted by an asterisk (*)

In addition to MOI, all barcodes with at least 5% proportion that StrainRecon reconstructed from the samples across all years were unique, 878 distinct strains in total. This finding suggests a lack of clonality in the Asembo Bay area.

Since the surveys 2007 and 2012 comprised individuals up to 20 years of age, a potential influence of age on MOI was further examined. There were no significant correlations between MOI and age, comparing children of 5 years and below with older ages, in 2007 (q = 0.07) or 2012 (q = 0.24) surveys, respectively.

In summary, the validation using field samples from Kenya suggests that the approach developed in this study could be used on the field samples for reliably reconstructing strains in individual samples and for detecting changes in MOI over time.

## Discussion

This study described the development of laboratory assays with multiplex PCRs followed by NGS, a unique bioinformatic process with B4Screening pathway and a novel threshold-calibrated MOI estimation method capable of detecting multiple-strain infections of *P. falciparum* parasites in artificially mixed laboratory strains and field isolates. Using this pipeline, the 24 barcode SNPs were identified successfully and uniformly from the 12 chromosomes of *P. falciparum* in a sample. The impact of pre-amplification, parasite concentration and strain proportion on the SNP frequency of mixed laboratory strains were evaluated. Parasite concentration within the tested range and pre-amplification did not influence the SNP frequency of strain within the same proportion, which allowed the evaluation of the field samples with a range of parasite densities with and without pre-amplification. Both DBS and frozen blood yielded sufficient reads in each SNP location analysed. Based on consistent barcode 24 SNP frequency calls at targeted locations, the algorithmic reconstruction of strains for each sample using a novel published StrainRecon [17] reconstructed the barcodes of dominant strains with 98.5% accuracy. In field samples from western Kenya, up to 5 strains in a sample were identified using same tools described above and temporal changes in 24 SNP barcode-based MOI could be reliably estimated using the novel threshold-calibrated STIM method developed in this study.

### Pipeline development

Multiplex PCR is a fast and cost-saving approach for pathogen diagnosis and genotyping [29, 30]. Three multiplex PCRs covering all 24 barcode SNPs identified by Daniels et al. [19] within the *P. falciparum* genome were developed in this study. The 24 short target regions needed to be amplified with comparable efficiency in multiplex PCRs to ensure generation of sufficient SNP coverage at each location via NGS. The 3 optimal multiplex PCRs were developed through a series of optimizations and pilot testing.

The most important and challenging issue was how to uniformly call the 24 barcode SNP frequencies based on NGS data and assign these frequencies to haplotypes. The second, interlinked, challenge was to determine how many strains were represented in each sample. To resolve these challenges, the StrainRecon mathematical algorithm for strain disambiguation [17] from different chromosomes and different SNPs [19] was leveraged in this study. It is important to point out that the consistency of SNP frequency calls at targeted SNP locations determines the ability of the StrainRecon and thus STIM to successfully assign these frequencies to haplotypes and disentangle the multiple strains within a sample and MOI estimation.

To maximize the consistency of SNP frequency calls from NGS data for haplotype assignment and strain reconstruction, the unique bioinformatics pipeline with B4Screeining pathway developed in this study first removed spurious amplicons introduced by sequencing. Initial data trimming and cleaning steps, by CutAdapt and Prinseq and data quality visualization via FaQCs prior to entry into the Bioconductor pipeline, ensured only sufficient quality, trimmed reads were processed. Without proper screening of noise, the extraneous element could erroneously generate orders of magnitude more sequences than truly exist in the data. There were numerous low copy number amplicons that did not reflect true diverse amplicons removed at population level using the novel and sensitive RF classifier developed in this study.

While this novel bioinformatics pipeline currently applies to the described 24-SNP malaria barcoding scheme, the implications of data cleaning steps here indicate the importance of careful evaluation of NGS output in any non-template-driven systems, such as whole genome multi locus sequence typing (wgMLST) or other barcoding approaches. The majority of unique amplicons (yet minority of total reads) generated in the laboratory and subsequently removed by routine bioinformatics processing did not reflect intended targets. In addition, chimerism between multiplex PCR targets was substantial in raw data. Therefore, leaving primers that are clearly distinct between sites on reads during the processing allowed a quick screen for chimeras. Incorporating this step prior to primer trimming also improves data quality and efficiency of analysis.

In the bioinformatics pipeline, where only information regarding known specific SNP sites (binary) was incorporated, the finding of multiple distinct point mutations per amplicon was not utilized. This latter information (category) can be incorporated in future work not only to enhance 24 SNP frequency calls but also to be useful for amplicon deep sequencing data analysis.

Although laboratory or clinical samples can have high parasite densities, field samples from population-based community surveys are collected largely from asymptomatic individuals who tend to have low parasite densities. In addition, in high and medium transmission areas, a minor proportion of parasite strains in a sample is often undetectable using conventional molecular technologies [31]. Both low parasite density samples and low proportion of parasite strains in a sample could increase difficulty in producing consistent frequencies across all SNP locations due to the limited DNA template availability. In this study, the impact of pre-amplification, parasite concentration and strain proportion on consistency of 24 SNP frequency calls were evaluated. It showed that SNP frequencies were not influenced by parasite concentration within the currently tested range or by pre-amplification, but differed by strain proportion. It is easy to envision that SNP frequency calls are entirely based on original DNA template diversity when noise and spurious amplicons from NGS are minimized using the unique bioinformatics developed in this study. Most importantly, the results have practical applications. First, it allows evaluation of field samples with a range of parasite densities, with and without pre-amplification. Second, pre-amplification allows evaluation of samples that have insufficient parasite concentration for analysis of diversity, but sufficient concentration to amplify without this step. Critically, NGS is sensitive for detection of low frequency SNPs in a sample [32, 33]. Compared to existing conventional molecular tools where minor parasite proportion below 10–30% in a sample were generally undetectable [9, 34–36], the tools developed in this study detected the barcodes of dominant strain with 98.5% accuracy and the proportion of parasite strains ranging from 2 to 10% in a sample with accuracy between 77.5 and 89.3%. Although the target lowest limit for minor strain detection was designed at 0.5 parasite/µl (see second subsection for laboratory assay development in Methods), this study did not attain such a fine level of resolution due to the inaccuracy observed at lower proportions (0.5–2%) of strains, specifically that as the strain proportion decreases, the ability to accurately detect the minor strain diminishes. This lower bound is influenced by both background noise level in the current analysis pipeline and the low DNA copy number of minor strains in the sample, decreasing expected precision. Nevertheless, the results from this study showed a substantial improvement in sensitivity for detecting minor parasite populations in a sample, particularly those above 5% proportion, with acceptable precision. While deep sequencing of individual targeted antigenic genes also provides ability to detect gene-specific minor variants in a sample, the estimates in the highly selected genes might not represent true genomic signatures of parasites [14, 15, 37] and may offer limited temporal and geographic discrimination between parasite populations [15].

The STIM algorithm for assessing MOI based on SNP read frequencies relies crucially on noise levels. Highly variable input in a noisy pipeline may cause MOI to be overestimated by confusing noise with true signal in reconstruction. A threshold was placed on MOI misfit, and calibrated by balancing the false negative rate of the strain reconstruction quality of StrainRecon on the known laboratory samples to the false positive rate and number of strains estimated. The same threshold was then used for running STIM on field data under the assumption that most noise would be from the pipeline steps against which the threshold already accounted. Importantly, the trends and conclusions from the field continue to hold even if the true threshold for this was slightly shifted relative to the one determined by the laboratory strain setting. In other words, MOI value estimated by STIM is subject to noise, whereas temporal changes in MOI as estimated by STIM are resilient to such noise. A large study across geographic regions is ongoing to examine the robustness of the STIM method in the field as well as potential needs for further calibration of the proposed threshold with a richer set of artificial strain mixtures.

### Field results

MOI has only recently been used as a metric for malaria transmission. Therefore, the EIR and malaria prevalence in children 5 years old and younger from same study area were obtained from 1995–1996 to 2012 (Fig. 5a, b, data extracted from both published [38] and unpublished KEMRI/CDC data) for side-by-side comparison with the MOI estimated from current study (Fig. 5c, d). The results show that the EIR sharply declined between 1995 and 2001 and remained low, even as malaria prevalence gradually decreased between 1996 and 2007, then reaching a plateau between 2007 and 2012. In comparison, the average MOI gradually declined over time and the percentage of samples with 5 strains dropped from 57% in 1996 to 18% in 2012 (during which period the proportion of one-strain samples increased). Since there was no correlation between MOI and age, the decline in MOI over time is unlikely to be confounded by host age [20]. Overall, the decreases in both average MOI and proportion of samples with 5 strains over time are in tandem with the decline in EIR and malaria prevalence; but the turning points are different. Specifically, MOI shows slow reduction, EIR has a sharp decline, and malaria prevalence stagnates from 2007 to 2012. This suggests a non-linear scaling relationship among the three malaria metrics [3]. The reasons behind the slow reduction in MOI are unclear; the large number of distinct strains detected in the area may play a role (878 distinct strains at least 5% proportion were detected in total and are reported in Results). Nevertheless, the MOI, which provides the information of strain numbers within a host, is a higher resolution parasite index compared with the malaria prevalence index and it might represent true transmission level. A further study of the parasite strain population size and strain relatedness is needed using this dataset.

The tools developed in this study advance both the estimation of number of strains within a host but also the number of strains at a population level, enhancing the resolution for MOI estimation. This advantage is particularly obvious compared to the original Taqman PCR 24 SNP barcode assay and COIL analysis for complexity of infection (COI) estimation in which monomorphic or polymorphic genotypes within each sample are estimated [19–21]. Taken together, the combined approach established in this study could be used for MOI estimation, particularly for temporal changes in MOI in regions with medium to high transmission levels. A large-scale validation study is being conducted using samples from different malaria countries/regions with heterogeneous transmission intensity.

## Conclusion

This study demonstrated that the combined approach of new multiplex PCRs, NGS and the unique bioinformatics pipeline developed in this study, together with the previously published StrainRecon algorithm, could identify prominent 24 barcode SNPs correctly and consistently across 12 of chromosomes of *P. falciparum*. Coupled with the novel threshold-calibrated MOI estimation method STIM, the proposed approach in this study provides a sensitive and high-resolution MOI estimator that could be used on field samples to measure temporal changes in MOI and to provide additional parasite indices for interpreting transmissions. The combined approach can potentially be used to study MOI in malaria clinical manifestations and to evaluate impact of interventions on transmission reduction for programme purpose. The utility of StrainRecon with the 24 SNP advanced laboratory tools and unique bioinformatics pipeline deserves further exploration for distinguishing recrudescence from re-infection in drug trials. Such tools can be further geared to tracking of imported malaria cases. The pseudocode for B4Screening pathway and STIM program code are available for download online at: https://www.ymsir.com/stim/.

## Supplementary Information

**Additional file 1: Figure S1.** RF classification using laboratory strains. Figure shows proportion of each unique amplicon relative to all amplicons from the targeted region on a plate from all laboratory strain samples based on RF classifier (when random selection determined the training set). X axis is the log_10_ count of SNP-specific reads in a Miseq run and y axis is log_10_ count of unique amplicon-specific reads in that individual run. Red points are amplicons classified as negative (False) for exclusion while green points are amplicons classified as positive (True) for further SNP frequency analysis.

**Additional file 2: Figure S2.** Depth of coverage across loci in laboratory strains. Log_10_ reads per SNP target location in laboratory strain data, from 1 to 24 IQR and outliers using a minimum threshold of 500 reads for a sample to be included. Green dots and right Y axis represent proportion of samples missing a value at each SNP site.

**Additional file 3: Figure S3.** Plot SNP23 from Combination A mixes. Plot SNP23 from Combination A mixes was made separately from all other SNPs. Data is also separated by source material (DBS and frozen blood samples). Within each mixture, left to right, the colors are: blue—all strains identical, orange—dominant strain unique, green—intermediate strain unique, yellow—low strain unique, and gray—SNP23 in Combination A mixes. A pilot experiment was also conducted using a different culture source, and this difference at SNP23 was not observed. Based on the consistency among all other SNP sites and the difference between parasite batches, SNP23 from Combination A was excluded from the Fig. 3 analysis concerning two distinct SNP reads.

**Additional file 4: Figure S4.** StrainRecon reconstruction on laboratory-mixed samples. Ground truth of barcodes (first row in each figure), the MAP estimate of the reconstruction matrix **M** and mixture vector ***v*** (second row), and the mean (third row) and standard deviation (fourth row) of the posterior density of candidate (**M**, ***v***) solutions. Each block contains one row for each strain, ordered by decreasing frequency from top, with a SNP cell color ranging from purple (fraction of 0) to bright yellow (fraction of 1). Each reconstruction is shown as the input parameter *n* (of the number of strains) is varied from *n* = 1 and 4 (row-major order) to be reconstructed in StrainRecon on the B_24NOPA_DBS sample. The dominant strain is captured perfectly and with high confidence by considering the posterior statistics. The algorithm has difficulty reconstructing the other two less-prevalent strains, since their target range of < 4% is low relative to the experimental noise levels seen in the pipeline.

**Additional file 5: Figure S5.** StrainRecon reconstruction on laboratory-mixed samples given advance knowledge of *n* = 3 strains. Ground truth of barcodes (first row in each figure), the MAP estimate of the reconstruction matrix **M** and mixture vector ***v*** (second row), and the mean (third row) and standard deviation (fourth row) of the posterior density of candidate (**M**, ***v***) solutions. Each block contains one row for each strain, ordered by decreasing frequency from top, with a SNP cell color ranging from purple (fraction of 0) to bright yellow (fraction of 1). The figure showcases the algorithm outputs with *n* = 3 strains on a variety of samples and mixtures, including cases of accurate and unique strain reconstruction (such as B33_PA_DBS).

## Data Availability

The source code for the B4Screening pathway for bioinformatics pipeline and STIM analysis tool is available online at: https://www.ymsir.com/stim/. All Sequence Read Archive (SRA) data for this study were submitted to NCBI BioProject under accession no. PRJNA555848, https://www.ncbi.nlm.nih.gov/sra/?term=PRJNA555848.

## Abbreviations

MOI: Multiplicity of infection
SNP: Single nucleotide polymorphism
NGS: Next generation sequencing
DBS: Dried blood spot
DNA: Deoxyribonucleic acid
PCR: Polymerase chain reaction
REF: Reference
ALT: Alternative
PA: Pre-amplification
NOPA: Non-pre-amplification
B4Screening: A novel pathway involving four new steps in bioinformatics pipeline
StrainRecon: A novel mathematical algorithm for haplotype analysis and Strain reconstruction
MAP: Maximum-a-posteriori
STIM: A novel mathematical algorithm for MOI estimation
IQR: Interquartile range
