# Supplementary figures and images for: Development of a new barcode-based, multiplex-PCR, next-generation-sequencing assay and data processing and analytical pipeline for multiplicity of infection detection of Plasmodium falciparum
Source: Malar J. 2021 Feb 16;20:92. doi: 10.1186/s12936-021-03624-2 (PMC7885407; doi:10.1186/s12936-021-03624-2)

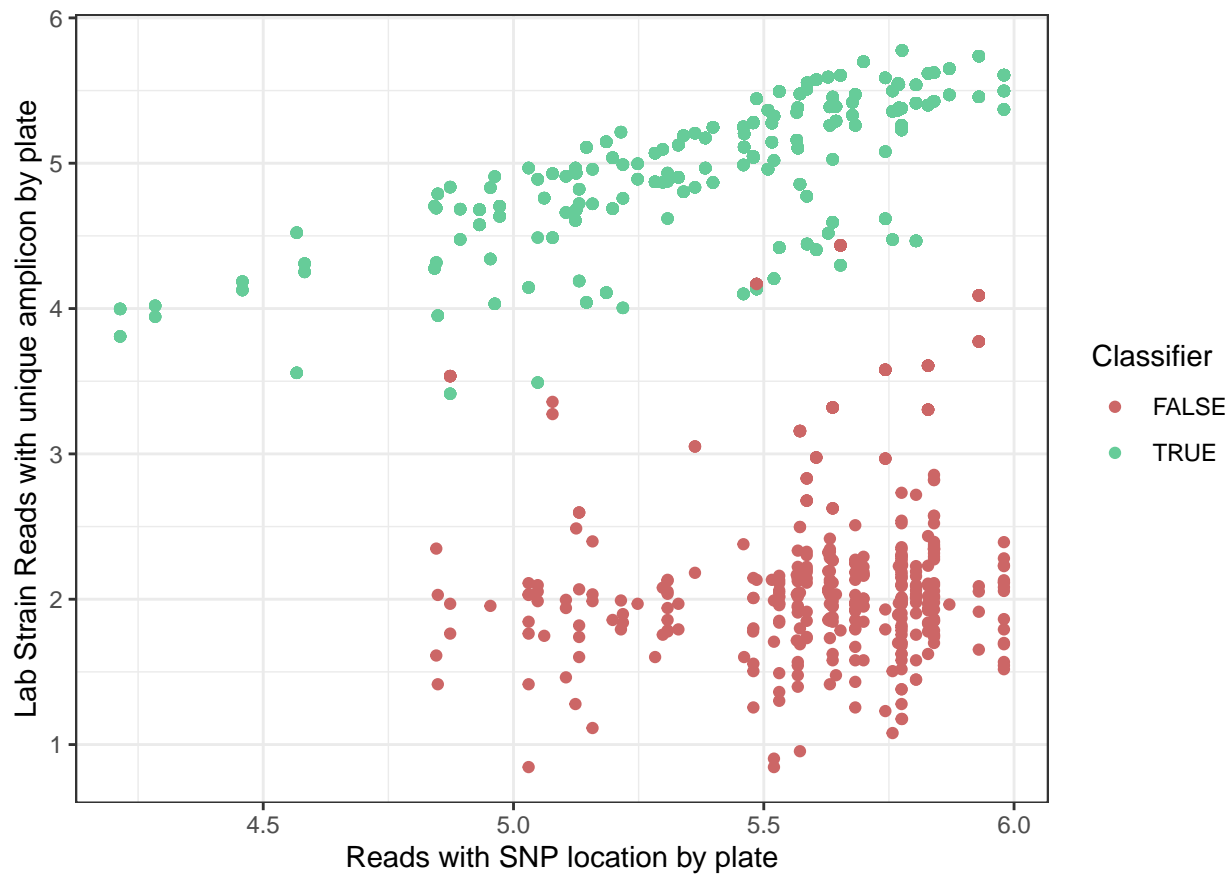

Supplement: Supplementary file 1 — Additional file 1: Figure S1. RF classification using laboratory strains. Figure shows proportion of each unique amplicon relative to all amplicons from the targeted region on a plate from all laboratory strain samples based on RF classifier (when random selection determined the training set). X axis is the log10 count of SNP-specific reads in a Miseq run and y axis is log10 count of unique amplicon-specific reads in that individual run. Red points are amplicons classified as negative (False) for exclusion while green points are amplicons classified as positive (True) for further SNP frequency analysis. [file 12936_2021_3624_MOESM1_ESM.pdf]

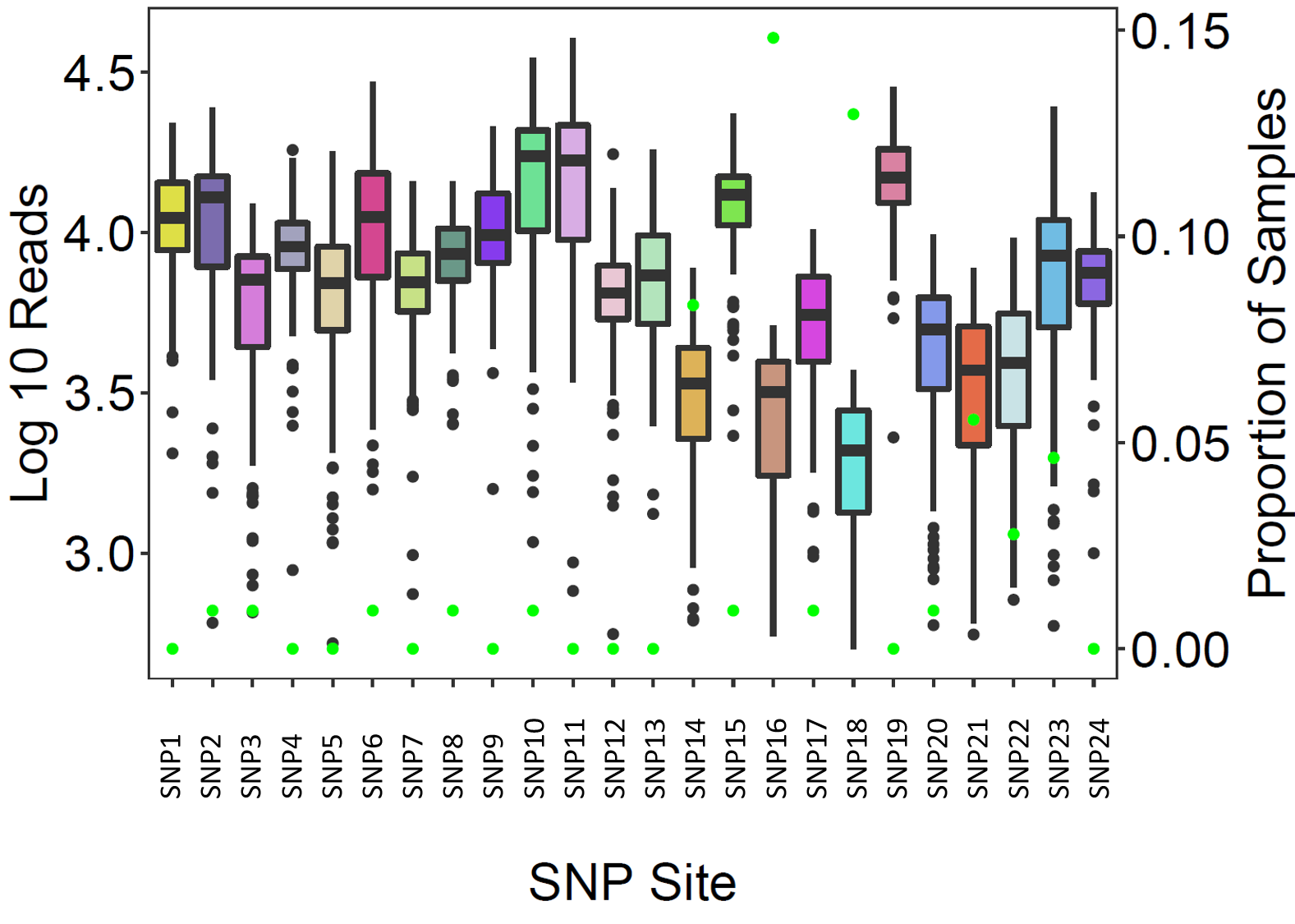

Supplement: Supplementary file 2 — Additional file 2: Figure S2. Depth of coverage across loci in laboratory strains. Log10 reads per SNP target location in laboratory strain data, from 1 to 24 IQR and outliers using a minimum threshold of 500 reads for a sample to be included. Green dots and right Y axis represent proportion of samples missing a value at each SNP site. [file 12936_2021_3624_MOESM2_ESM.tif]

# Scatter of all accepted points

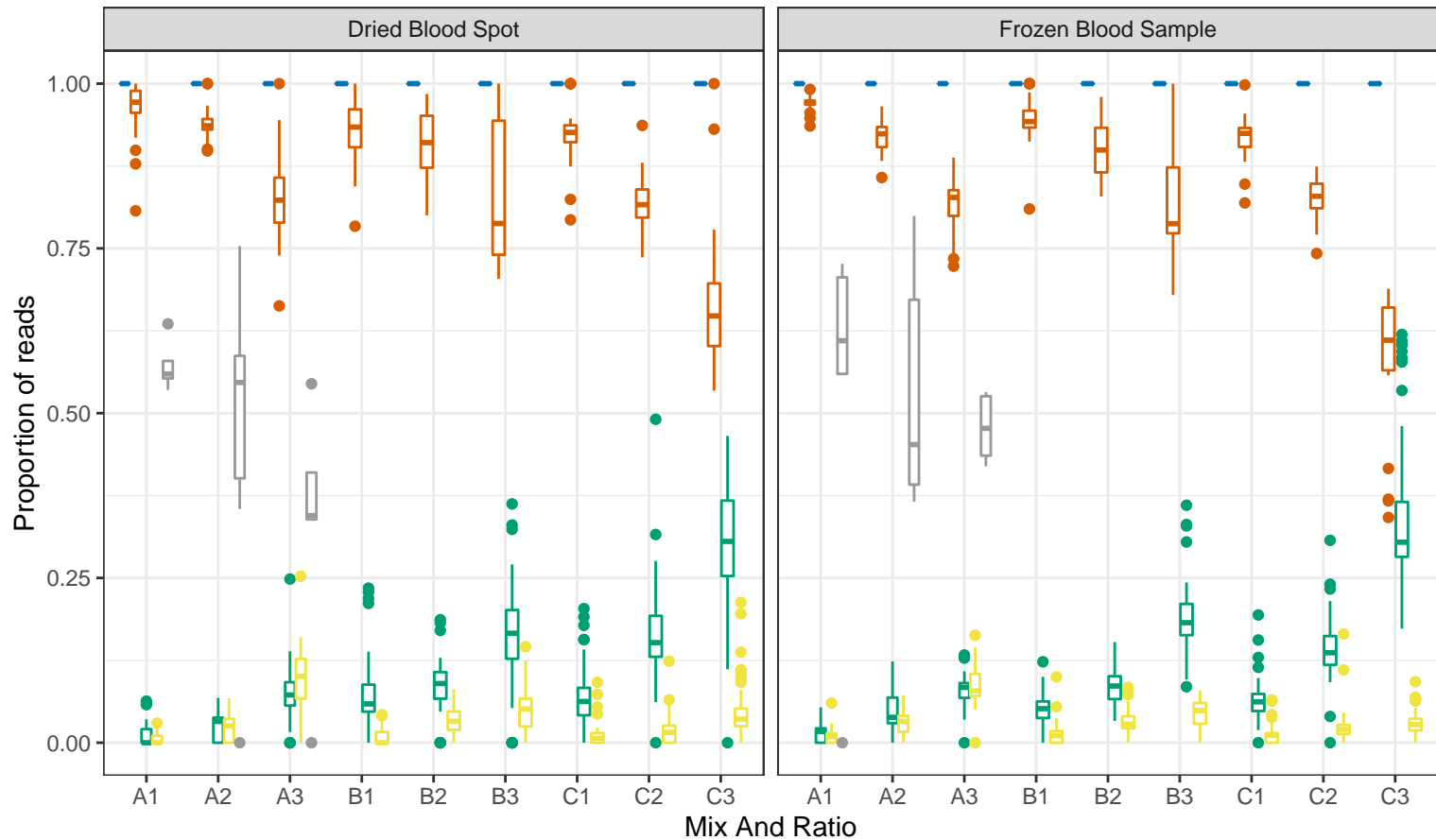

Supplement: Supplementary file 3 — Additional file 3: Figure S3. Plot SNP23 from Combination A mixes. Plot SNP23 from Combination A mixes was made separately from all other SNPs. Data is also separated by source material (DBS and frozen blood samples). Within each mixture, left to right, the colors are: blue—all strains identical, orange—dominant strain unique, green—intermediate strain unique, yellow—low strain unique, and gray—SNP23 in Combination A mixes. A pilot experiment was also conducted using a different culture source, and this difference at SNP23 was not observed. Based on the consistency among all other SNP sites and the difference between parasite batches, SNP23 from Combination A was excluded from the Fig. 3 analysis concerning two distinct SNP reads. [file 12936_2021_3624_MOESM3_ESM.pdf]

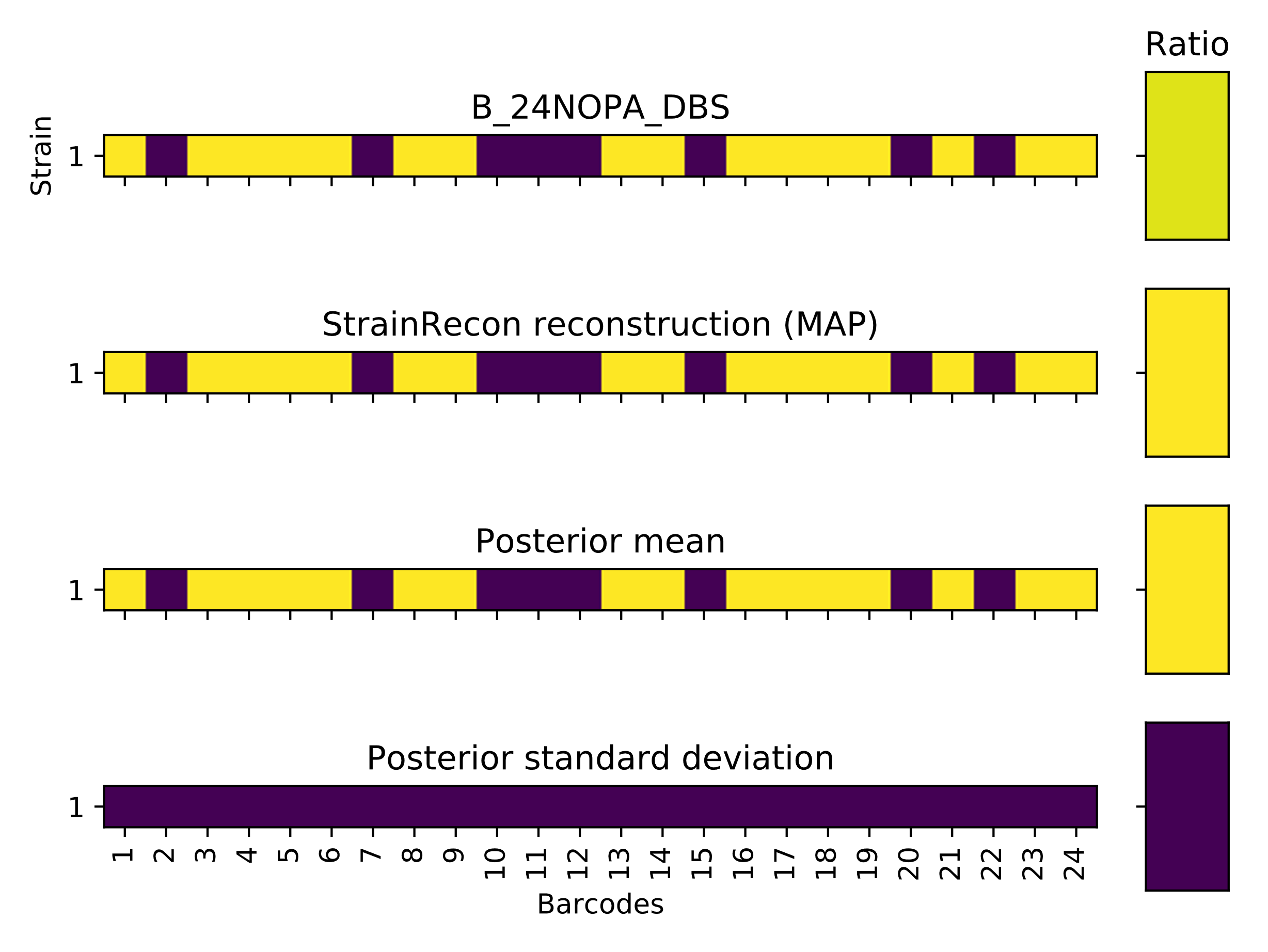

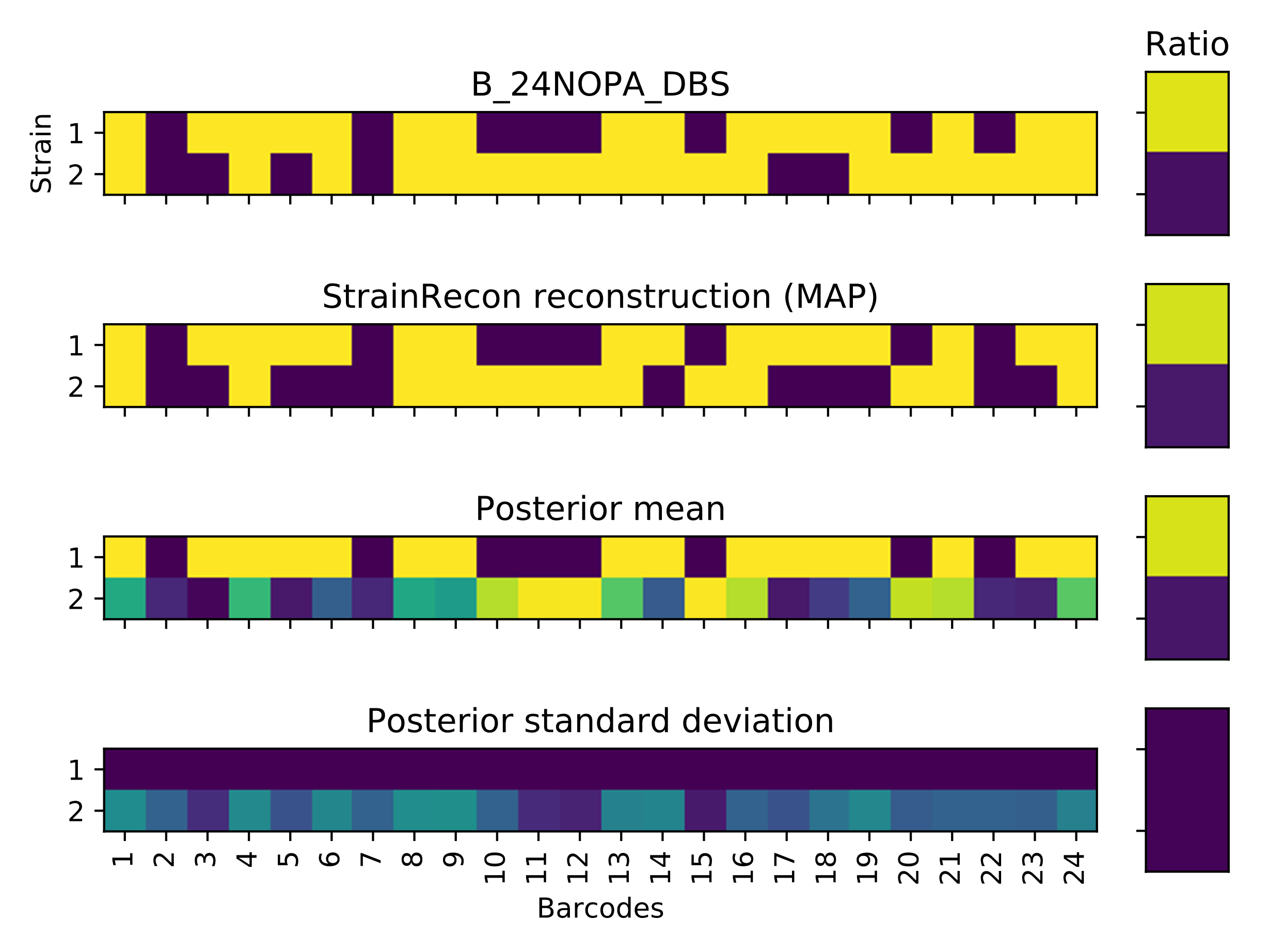

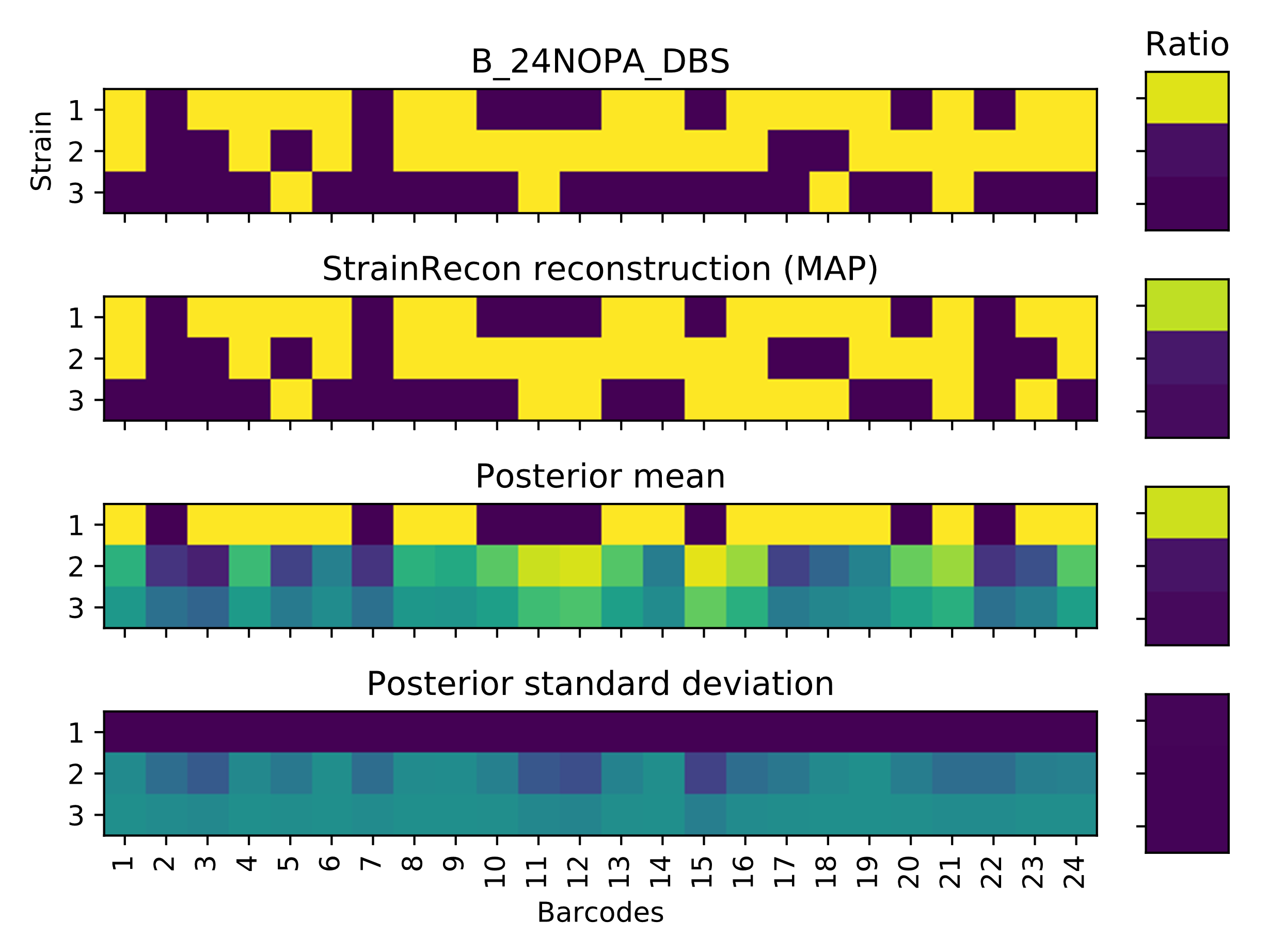

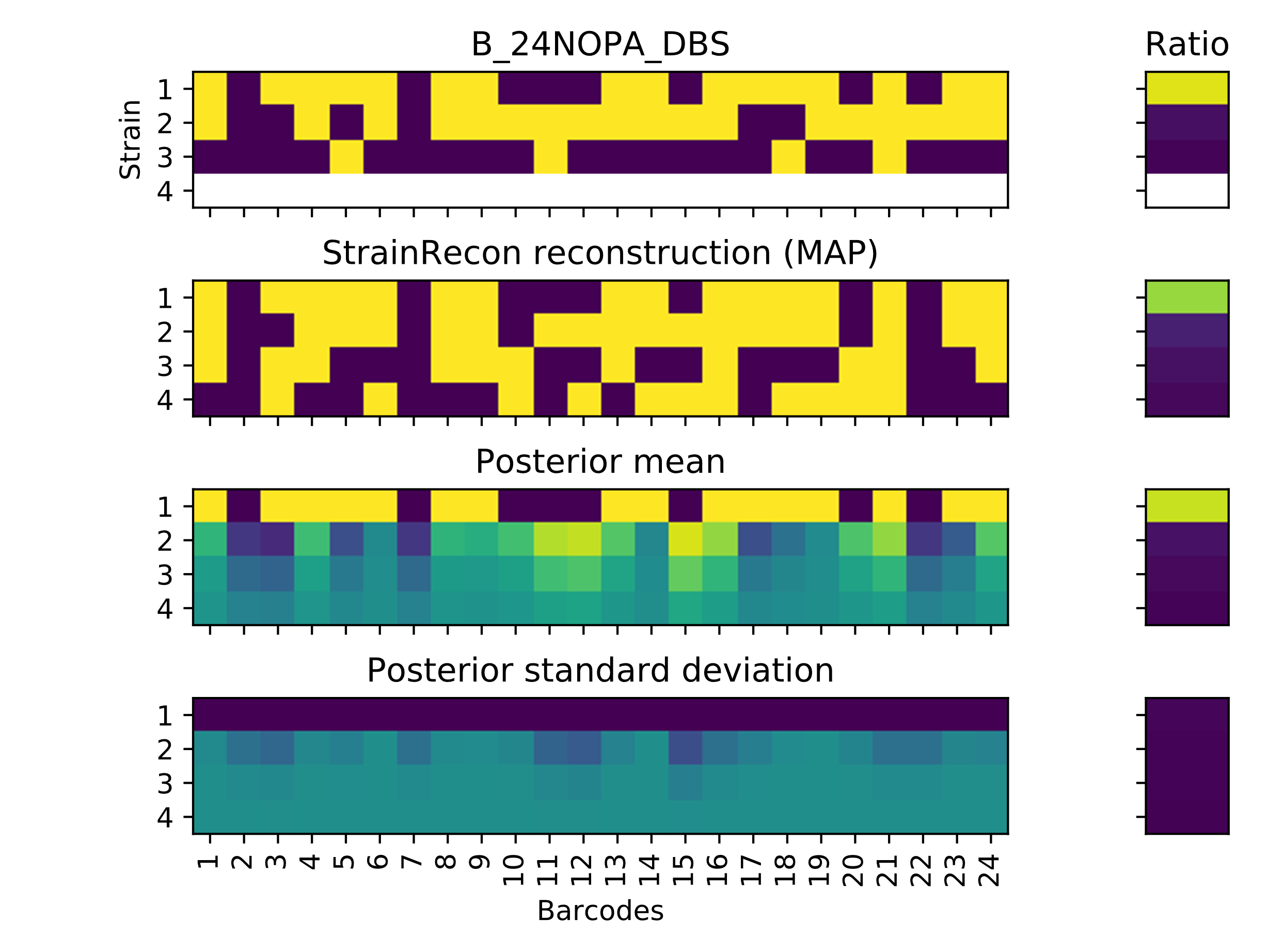


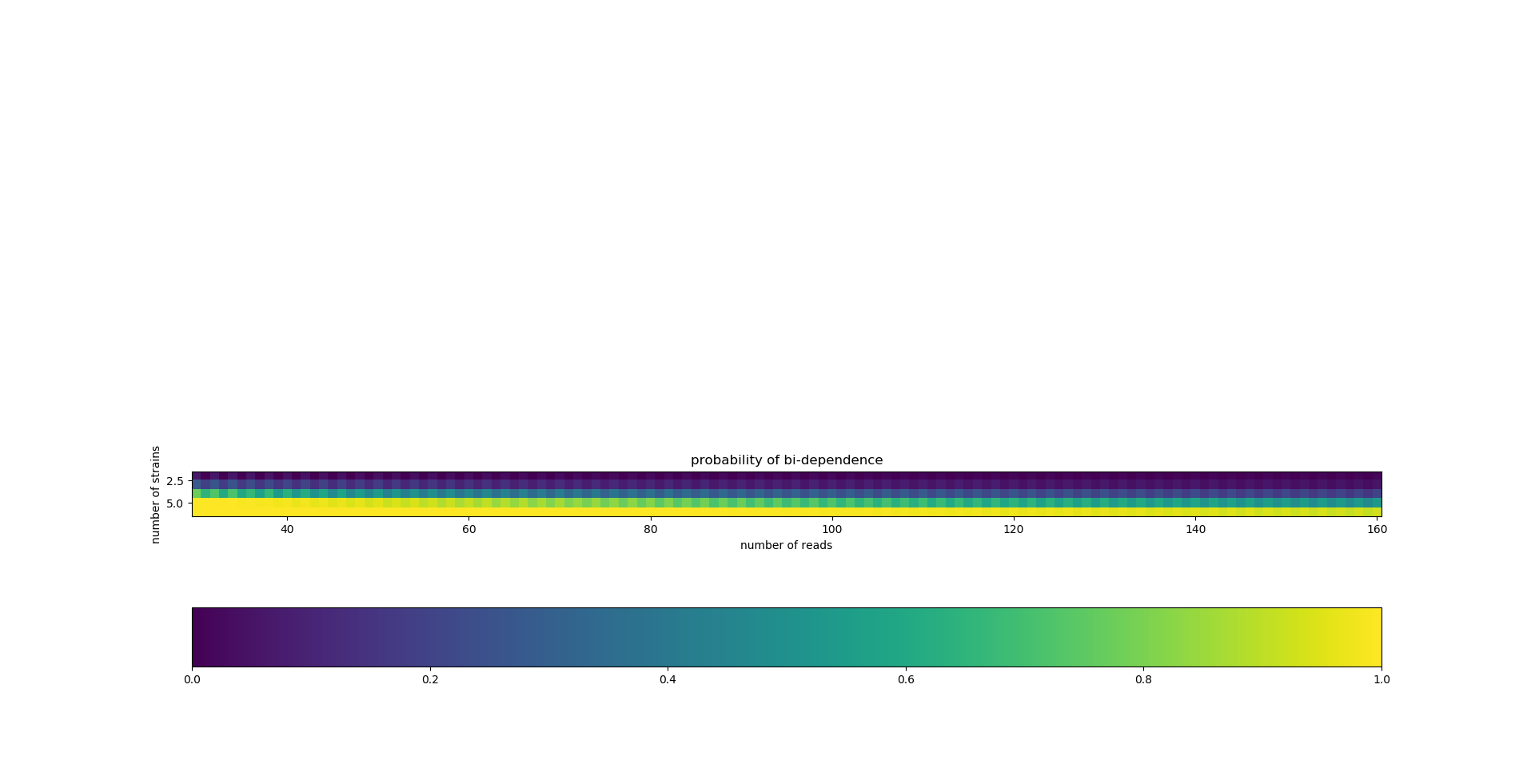

Supplement: Supplementary file 4 — Additional file 4: Figure S4. StrainRecon reconstruction on laboratory-mixed samples. Ground truth of barcodes (first row in each figure), the MAP estimate of the reconstruction matrix M and mixture vector v (second row), and the mean (third row) and standard deviation (fourth row) of the posterior density of candidate (M, v) solutions. Each block contains one row for each strain, ordered by decreasing frequency from top, with a SNP cell color ranging from purple (fraction of 0) to bright yellow (fraction of 1). Each reconstruction is shown as the input parameter n (of the number of strains) is varied from n = 1 and 4 (row-major order) to be reconstructed in StrainRecon on the B_24NOPA_DBS sample. The dominant strain is captured perfectly and with high confidence by considering the posterior statistics. The algorithm has difficulty reconstructing the other two less-prevalent strains, since their target range of < 4% is low relative to the experimental noise levels seen in the pipeline. [file 12936_2021_3624_MOESM4_ESM.docx]

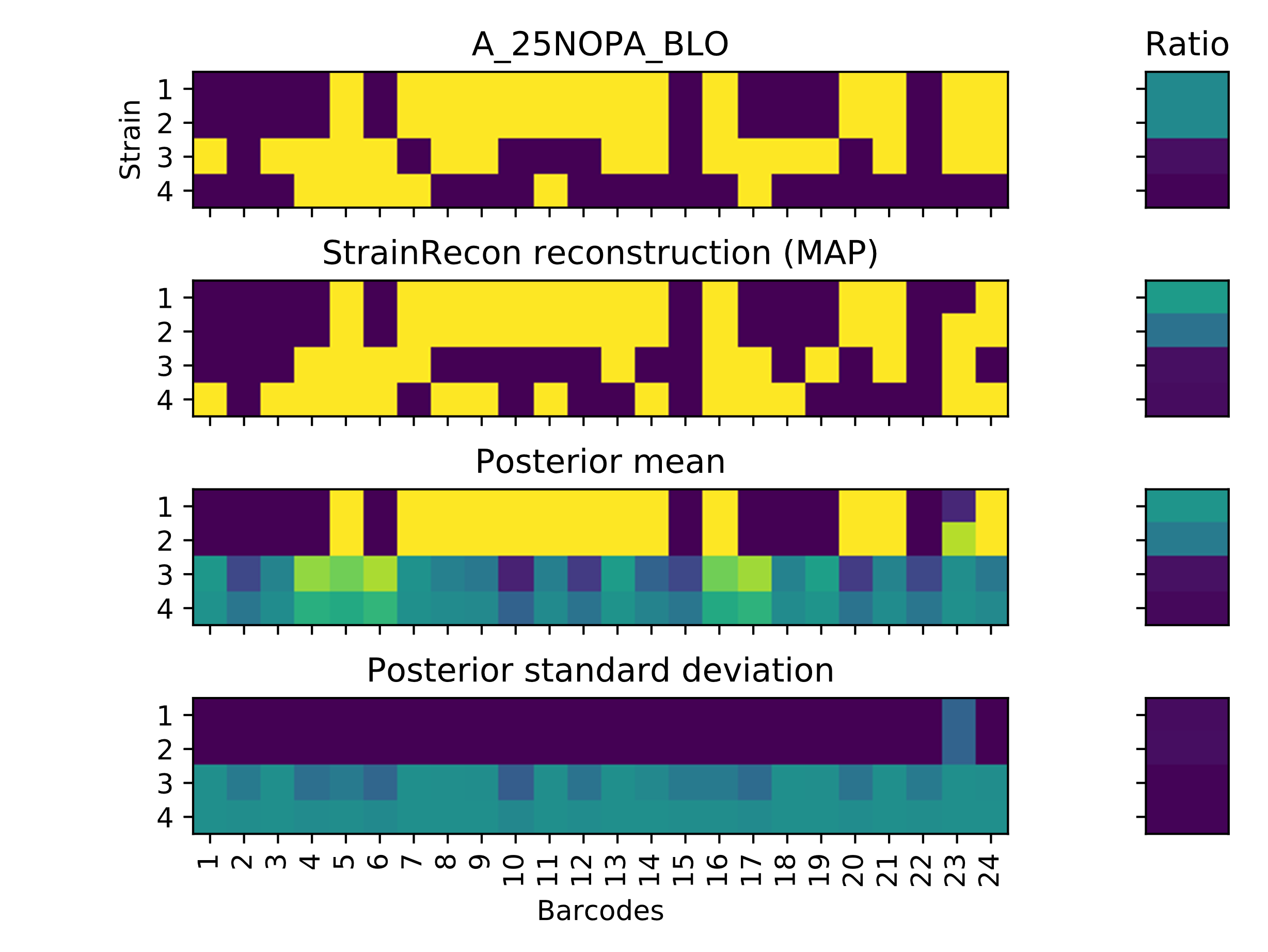

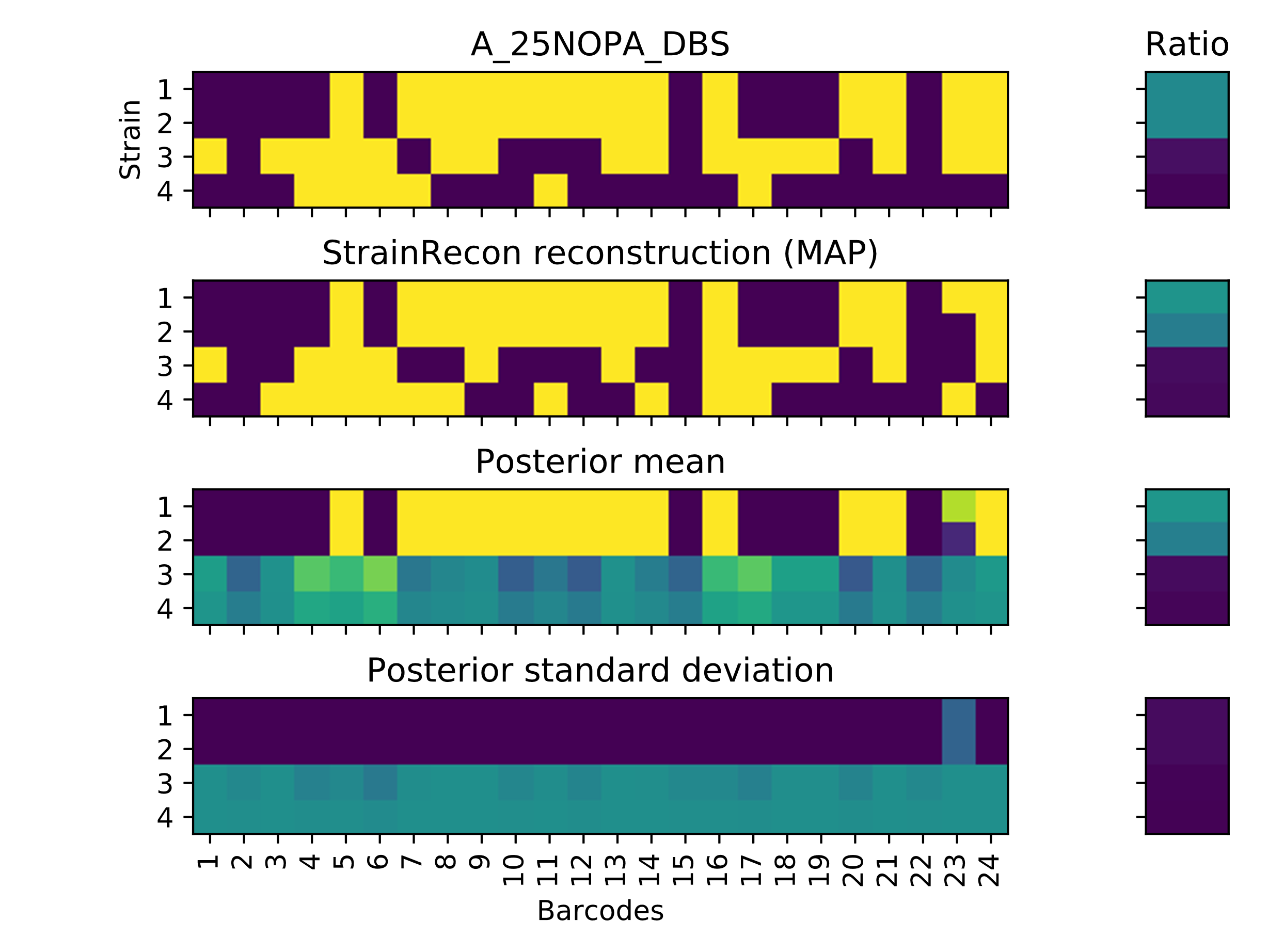

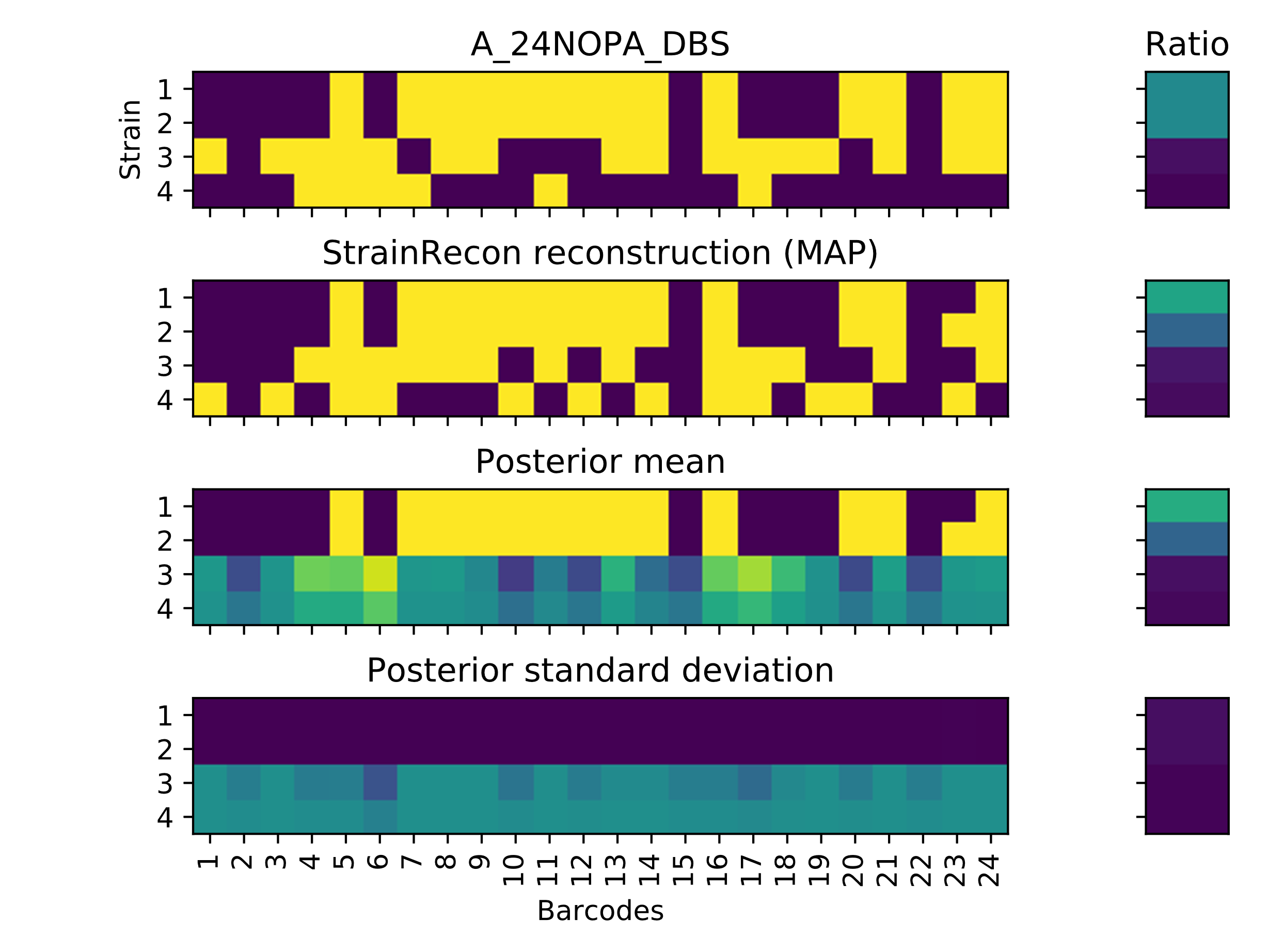

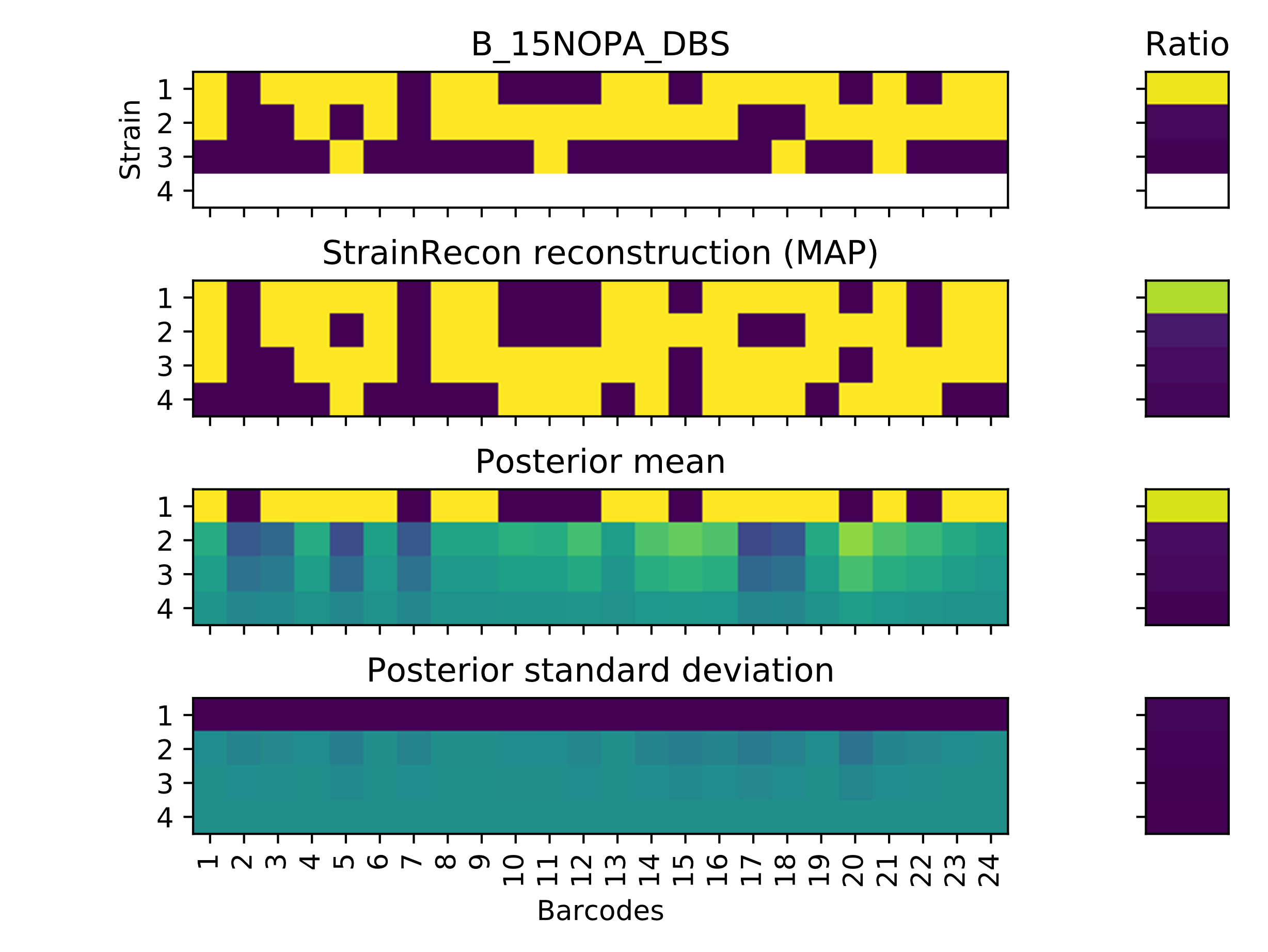

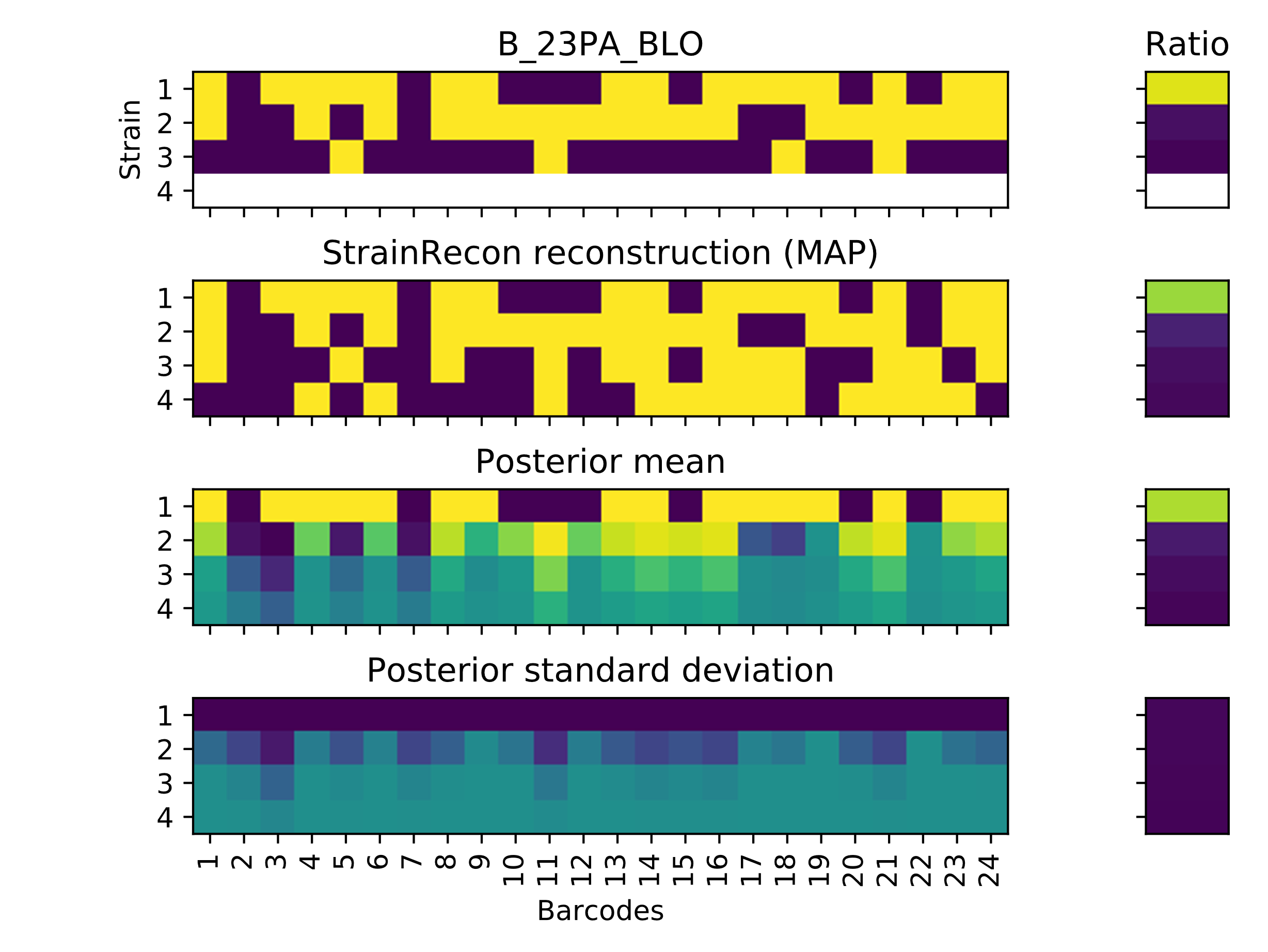

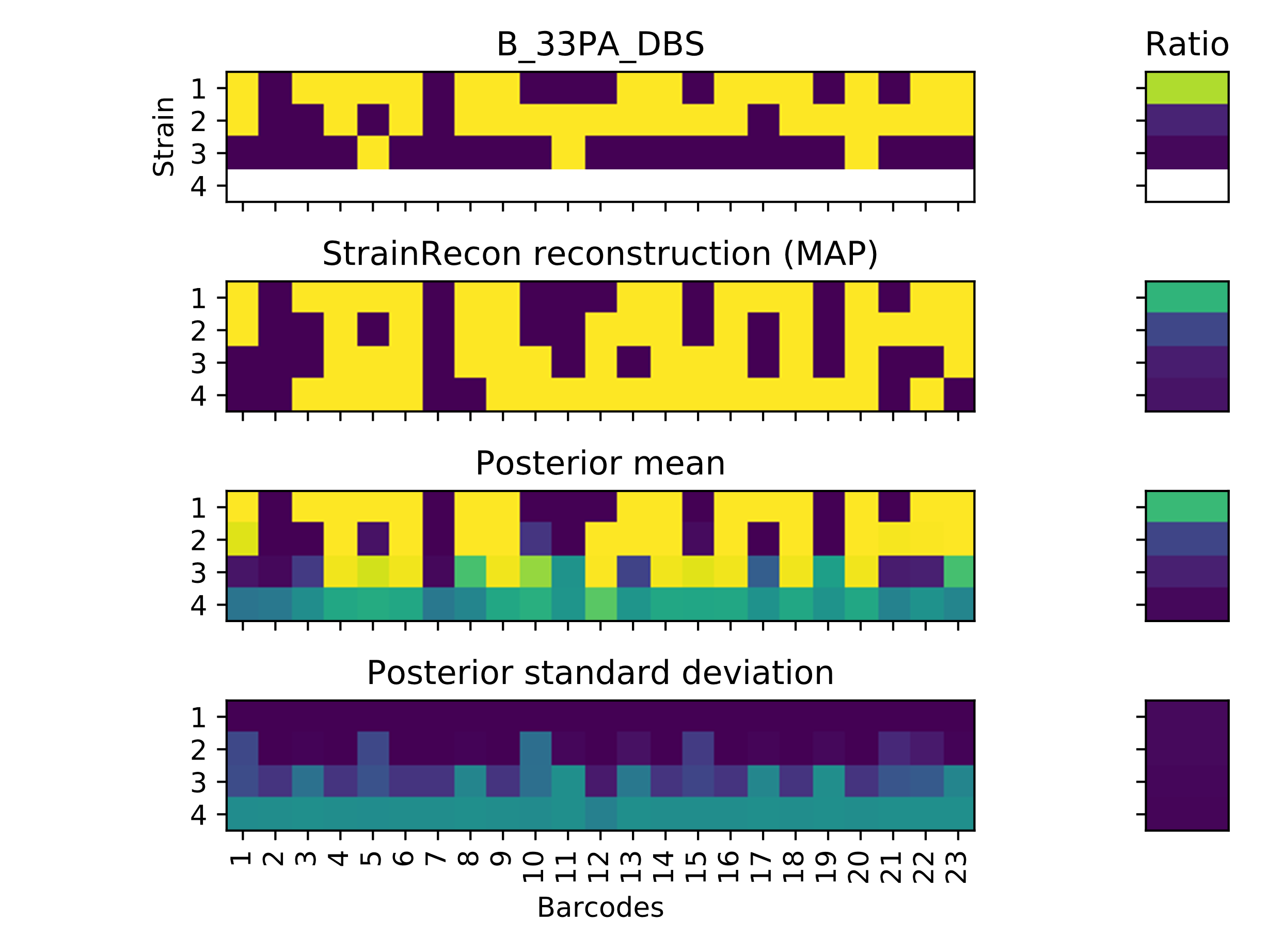

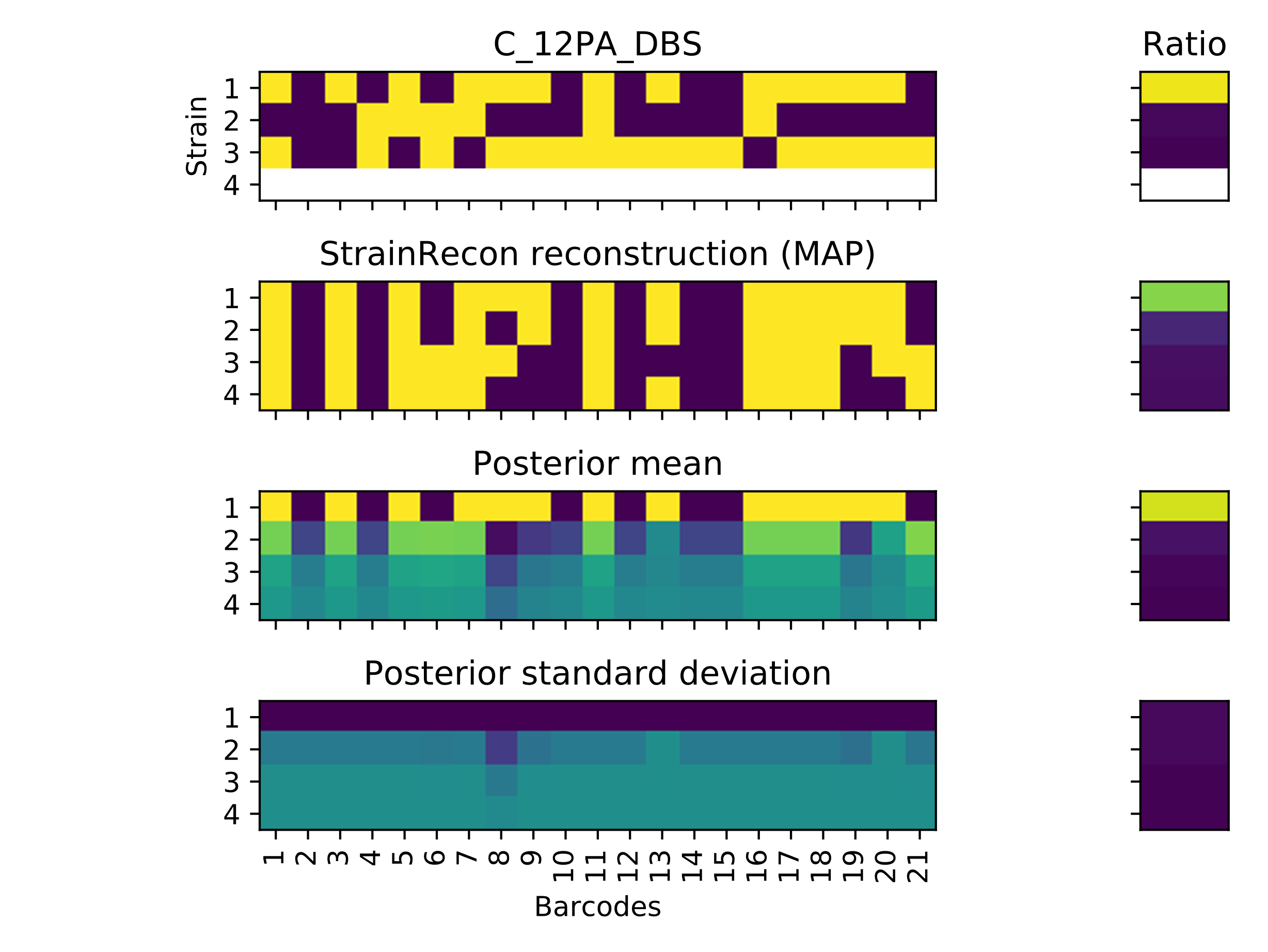

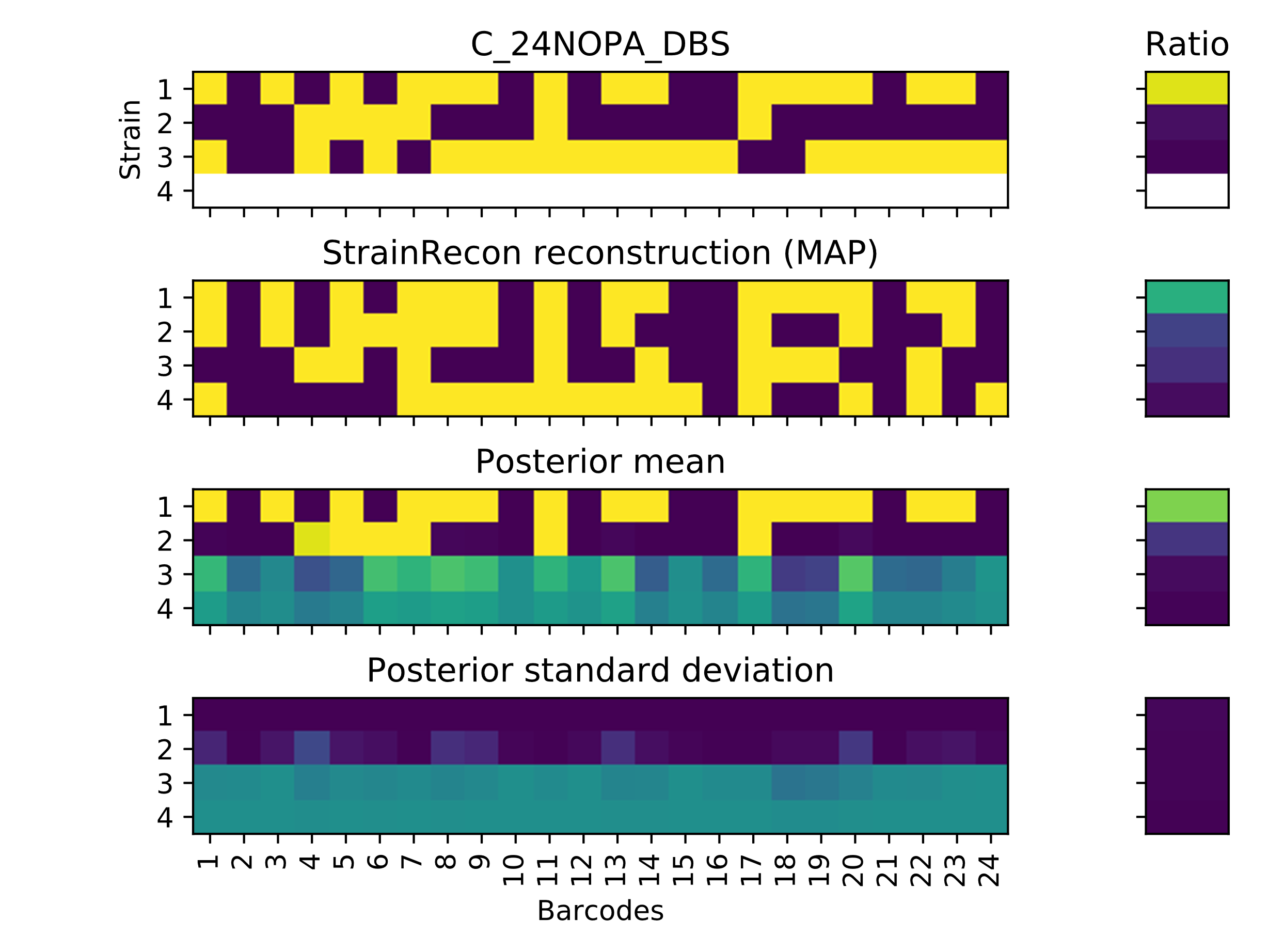

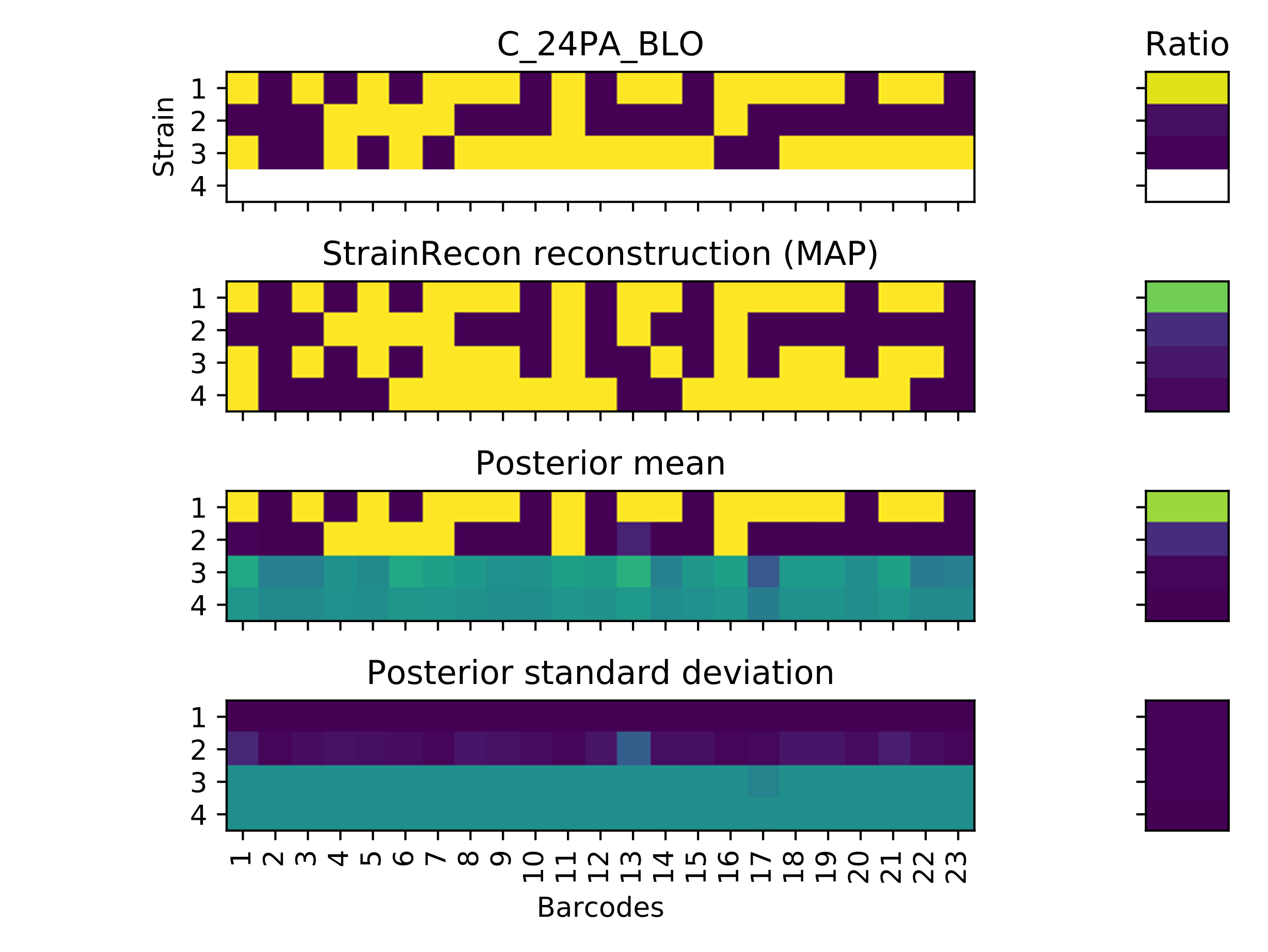

Supplement: Supplementary file 5 — Additional file 5: Figure S5. StrainRecon reconstruction on laboratory-mixed samples given advance knowledge of n = 3 strains. Ground truth of barcodes (first row in each figure), the MAP estimate of the reconstruction matrix M and mixture vector v (second row), and the mean (third row) and standard deviation (fourth row) of the posterior density of candidate (M, v) solutions. Each block contains one row for each strain, ordered by decreasing frequency from top, with a SNP cell color ranging from purple (fraction of 0) to bright yellow (fraction of 1). The figure showcases the algorithm outputs with n = 3 strains on a variety of samples and mixtures, including cases of accurate and unique strain reconstruction (such as B33_PA_DBS). [file 12936_2021_3624_MOESM5_ESM.docx]
